# A General Definition and Nomenclature for Alternative Splicing Events

**DOI:** 10.1371/journal.pcbi.1000147

**Published:** 2008-08-08

**Authors:** Michael Sammeth, Sylvain Foissac, Roderic Guigó

**Affiliations:** Centre de Regulació Genòmica, Barcelona, Spain; Washington University, United States of America

## Abstract

Understanding the molecular mechanisms responsible for the regulation of the transcriptome present in eukaryotic cells is one of the most challenging tasks in the postgenomic era. In this regard, alternative splicing (AS) is a key phenomenon contributing to the production of different mature transcripts from the same primary RNA sequence. As a plethora of different transcript forms is available in databases, a first step to uncover the biology that drives AS is to identify the different types of reflected splicing variation. In this work, we present a general definition of the AS event along with a notation system that involves the relative positions of the splice sites. This nomenclature univocally and dynamically assigns a specific “AS code” to every possible pattern of splicing variation. On the basis of this definition and the corresponding codes, we have developed a computational tool (AStalavista) that automatically characterizes the complete landscape of AS events in a given transcript annotation of a genome, thus providing a platform to investigate the transcriptome diversity across genes, chromosomes, and species. Our analysis reveals that a substantial part—in human more than a quarter—of the observed splicing variations are ignored in common classification pipelines. We have used AStalavista to investigate and to compare the AS landscape of different reference annotation sets in human and in other metazoan species and found that proportions of AS events change substantially depending on the annotation protocol, species-specific attributes, and coding constraints acting on the transcripts. The AStalavista system therefore provides a general framework to conduct specific studies investigating the occurrence, impact, and regulation of AS.

## Author Summary

The genome sequence is said to be an organism's blueprint, a set of instructions driving the organism's biology. The unfolding of these instructions—the so-called genes—is initiated by the transcription of DNA into RNA molecules, which subsequently are processed before they can take their functional role. During this processing step, initially identical RNA molecules may result in different products through a process known as alternative splicing (AS). AS therefore allows for widening the diversity from the limited repertoire of genes, and it is often postulated as an explanation for the apparent paradox that complex and simple organisms resemble in their number of genes; it characterizes species, individuals, and developmental and cellular conditions. Comparing the differences of AS products between cells may help to reveal the broad molecular basis underlying phenotypic differences—for instance, between a cancer and a normal cell. An obstacle for such comparisons has been that, so far, no paradigm existed to delineate each single quantum of AS, so-called AS events. Here, we describe a possibility of exhaustively decomposing AS complements into qualitatively different groups of events and a nomenclature to unequivocally denote them. This typological catalogue of AS events along with their observed frequencies represent the AS landscape, and we propose a procedure to automatically identify such landscapes. We use it to describe the human AS landscape and to investigate how it has changed throughout evolution.

## Introduction

Alternative splicing (AS) is a fundamental molecular process regulating eukaryotic gene expression and involved in numerous human diseases [1]–[3]. It is usually postulated as the main mechanism to augment protein diversity from a somehow limited set of protein coding genes [4]. Consequently, over the recent years various large scale studies have been undertaken aiming at the exhaustive identification and analysis of AS events (for recent reviews, see [5]–[7]). Current estimations claim around 60–75% of human multi-exonic genes to undergo AS [4],[8],[9].

**Figure 1:**
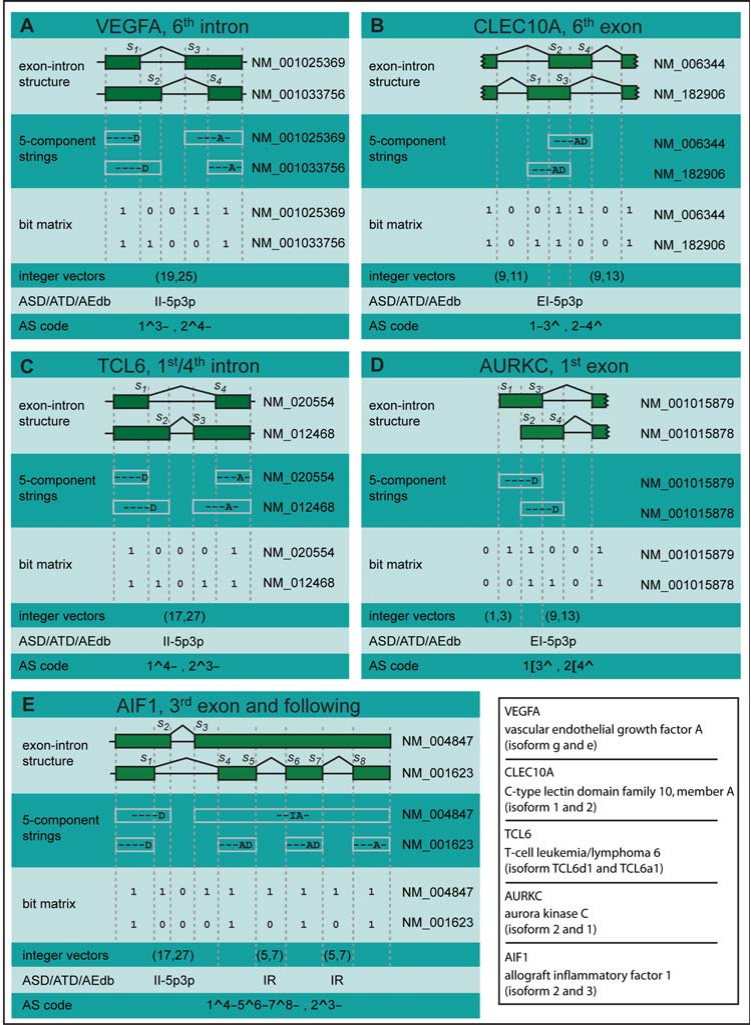
Comparison of nomenclatures for alternative splicing. Examples of splicing structures in the 5 human genes VEGFA (A), CLEC10A (B), TCL6 (C), AURKC (D), and AIF1 (E). In each case a schema of the exon–intron structure is shown where variable sites 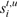 are numbered consecutively from 5′ to 3′. Subsequently, the splicing structure is described with the Malko's 5-component strings, Nagasaki's bit matrices and integer vectors, the nomenclature of the ASD/ATD/AEdb databases and with the AS code we propose in this work. The nomenclature of ASD/ATD/AEdb assigns ambiguously the same identifier to the structures in VEGFA (A) and TCL6 (C), respectively in CLEC10A (B) and AURKC (D). In CLEC10A (B), the bit matrix system assumes independence between both sides of the exon and therefore can not identify a single AS event. In AURKC (D), the vector (1,3) is assignable from the bit matrices, but it is not considered as part of the alternative donor event (9,13). Authors of the ASD/ATD/AEdb nomenclature propose the term “CIR” for complex intron retention structures. However, as in AIF1 (E), the selection of the central intron can be problematic as the names “CIR-II-5p3p-5p-IR-3p”, “CIR-CIR-II5p3p-5p-5p”, or “CIR-II5p4p-CIR-IR-3p-3p” could be imaginable.

Surprisingly, to some extent, the rigorous formalization of the concept of AS event and its categorization has received relatively little attention. Traditionally, terms for only five kinds of AS events have been coined: exon skipping (ES), mutually exclusive exons (ME), intron retention (IR), alternative donor (AD) and acceptor (AA) sites [10]. However, currently available transcript evidence shows a plethora of variations in splicing patterns that involve multiple instances of these classical events in various combinations [11]. Figure 1 and Figure S1 give some examples of AS patterns observed in the manually curated RefSeq annotation [12]. Despite the ever growing availability of gene annotations the lack of a universal reference definition of AS and hence of the corresponding categories of AS events prevent AS databases (e.g., AEdb [13], ASD [14], ATD [15], Hollywood [16], PASDB [17], SpliceNest [18], PALS db [19], SpliceDB [20], AsMamDB [21], HASDB [22], ProSplicer [23], EuSplice [24], ASAPII [25] etc. …), from the automatic identification and update of the AS landscape that characterizes the transcriptome from a particular cell type or condition. Such a specific landscape may be revealing the underlying biological mechanisms responsible for the cell's phenotype. Towards that end the challenges to be addressed are (i) to define and identify single instances of AS events in complex exon–intron variations, (ii) to find an intuitive vocabulary to adequately characterize different AS events, and (iii) to develop methods to efficiently identify and classify AS events from sets of annotated transcripts.

Concerning challenge (i) Malko and co-workers proposed to combine the classical terms for each exon observed in a given annotation [26]. While variations of each exon across the compared transcripts can be sufficiently described by this procedure, it does not permit an easy extension for splicing variations across the adjacent introns. However, some splicing evidence (e.g., the structures depicted in Figure 1A and 1C) suggests a relation between splice sites across the intron as by means of intron definition mechanisms [27],[28]. In another study, Nagasaki *et al.* propose to delineate AS events at exonic regions common to the compared transcripts [29]. Whereas in this approach intron-defined splicing variations are coherently modelled, events that could be connected by exon definition [30] are by definition assumed to be independent and are treated separately (as for instance in Figure 1B). Furthermore, the separation at overlapping exonic positions does not allow for investigation of potential relations between transcription and splicing, i.e., relative position of the initiation and polyadenylation site (Figure 1D), for which increasing evidence is reported in literature [31],[32]. The EnsEmbl databases on splicing, transcript, and exon diversity (ASD, ATD, and AEdb) have recently extended their definition of AS events (e.g., “complex intron retention”) in order to allow for additional modifications upstream and/or downstream of a central event. However, this notation system still remains limited and fails to capture structures depicted in Figure 1E or larger.

Addressing problem (ii), only few attempts have been undertaken to univocally denote AS events. Malko et al. [26] proposed strings composed of 5 letters identifying each classical event to redundantly describe the variability separately for each exon observed in a certain annotation (e.g., “—AD” for combined variable acceptor and donor sites, Figure 1B). These 5-component strings naturally bear a high degree of redundancy as one is required for each different form of exon. Furthermore, the picture of the exon–intron structure can not be inferred solely from these strings, as can be seen by the structures in Figure 1A and 1C producing identical results. Nagasaki and co-workers proposed the so-called “bit matrices”, binary matrices to describe AS events where each row represents a transcript variant and each column represents a genomic position. Each position of the matrix is filled by “1” and “0” according to whether the respective transcript variant exhibits an exon or not at the corresponding position. Neighboring identical columns then are collapsed, such that variations in the exon–intron structure are represented non-redundantly as flip-flop changes. This representation draws a pictorial “bitmap” of the exon–intron structure from compared transcripts. Disadvantages are that the number of “bits” that have to be shown (i.e., the matrix area) is relatively large even for simple events (e.g., 14 for the event in Figure 1B). Therefore, a condensed encoding of the bits in 2-dimensional integer vectors has been proposed, which however looses transparency of the exon–intron structure. Alternatively, the nomenclature of ASD/ATD/AEdb focuses up to a certain degree on the location of variations around a centric intron/exon up to a certain degree, but does not describe the relative connection between these variations. For instance, a name as “II-5p3p” (i.e., “intron isoform with modification at the 3′- and 5′-end”) cannot distinguish the cases depicted in Figure 1A and 1C. Correspondingly, the term “EI-5p3p” is ambiguous considering the structures in Figure 1B and 1D. The number of such ambiguities grows with the number of concatenated terms: four different splicing structures for instance match the term “CIR-EB-5p3p”. Also, the identification of a “central event” becomes problematic in large splicing variations (Figure 1E).

With respect to issue (iii), splicing graphs as a non-redundant data structure have gained popularity in AS over the recent years, but definitions vary across literature. Capturing the 5′→3′ directionality of transcription, they naturally all form directed acyclic graphs (DAGs). Going back to [33], matching (parts of) ESTs [22],[34],[35] have been used as nodes connected by edges representing the EST evidence, in order to cluster them and/or to allow the analysis of AS. Heber and co-workers [35] subsequently collapse (remove) vertices with *indegree* (i.e., the number of inedges) = *outdegree* (the number of outedges) = 1. Later on, two works from the same year proposed a graph structure where every vertex corresponds to a splice site and the connecting edges represent the intermediate exon/intron [36],[37], labelled according to the mRNA or EST evidence. Another kind of graph uses exons as nodes instead of splice sites [38]. Whereas intuitive for visualization, the graph structure may redundantly contain common exon flanks. Other graph-based approaches on exon–intron structures described in literature use similar techniques [25], [39]–[41]. However, all these analyses focus exclusively on the four types of traditional AS events, and thus capture only a limited fraction of the splicing variation encompassed in the transcriptome—probably a main consequence of problem (i). Indeed, without a universal definition of AS event, the retrieval of a single type of splicing variation requires to define its corresponding sub-graph pattern and to localize all occurrences of this pattern in the whole splicing graph. Consequently, a comprehensive characterization of AS needs an exhaustive set of such ad hoc patterns, which explains why usually only 4–6 types of events are considered.

In this work, we propose a general definition of “AS event” and we present a novel notation based on the relative position of alternative exon boundaries to flexibly describe such events. Unlike traditional nomenclatures, this generic notation system allows the assignment of a univocal “AS code” to identify any possible variation of the exon–intron structure between two or more transcripts, and thus provides a platform for the automatic and exhaustive extraction of such variations from a dataset of annotated genes. Here, we also describe in detail the method implemented in AStalavista (Alternative Splicing transcriptional landscape visualization tool) for the dynamic characterization of AS events in splicing graphs. AStalavista is accessible as a web server at (http://genome.imim.es/astalavista) [42]. We have used AStalavista to characterize and compare the “landscape” of AS in different human reference annotations as well as in annotations of other metazoan species, i.e., chimp (*Pan troglodytes*), mouse (*Mus musculus*), rat (*Rattus norvegicus*), dog (*Canis familiaris*), cow (*Bos taurus*), chicken (*Gallus gallus*), frog (*Xenopus tropicalis*), zebrafish (*Danio rerio*), honeybee (*Apis mellifera*), fruitfly (*Drosophila melanogaster*), and worm (*Caenorhabditis elegans*). In contrast to previous large-scale studies, our approach focuses on splicing structure variations rather than on (sequence) attributes of alternative exons/introns [43],[44]. Results indicate that while most AS events can be assigned to a few categories, the categorization of AS events in different structures is quite complex, with a plethora of minor AS configurations. Relative frequencies of particular patterns change with respect to the corresponding annotation protocol, species-specific attributes and coding constraints of the respective locus, and we present computational studies that investigate the reasons behind these fluctuations.

## Results

### A General Definition of AS Event

The concurrent and regulated molecular mechanisms of exon and intron definition are generally responsible for the splicing structure in a certain transcript variant. Although case studies for the mechanics of intron and exon recognition are given in literature [27],[28],[30], no general rule could (yet) be deduced. Therefore, neither of the mechanisms can be excluded from occurring during the splicing process and both are to be considered in a generally robust definition of AS event that is applicable to any organism without being *a priori* restricted to exon or intron definition. In order to allow for possible interactions of parts of the splicing machinery across all exons and introns when delimiting AS events in exon–intron variations, our definition of AS events is based on *sites*: given an annotation, i.e., transcript sequences aligned to the genome, we use the terminus “site” to describe genomic locations of aligned exon boundaries (Definition 1).

#### Definition 1 (Site)

A site *s* is an exon boundary as characterized by its genomic position *pos*(*s*) and its type *type*(*s*) to distinguish between transcription start sites (TSS) *type*(*s*) = *σ*, splice donors *type*(*s*) = *δ*, splice acceptors *type*(*s*) = *α* and polyadenylation sites (PAS) *type*(*s*) = *ω*. Each site is supported by a set of transcripts *transcripts*(*s*) that all show evidence for *s* in the annotated exon–intron structure.

**Figure 2:**
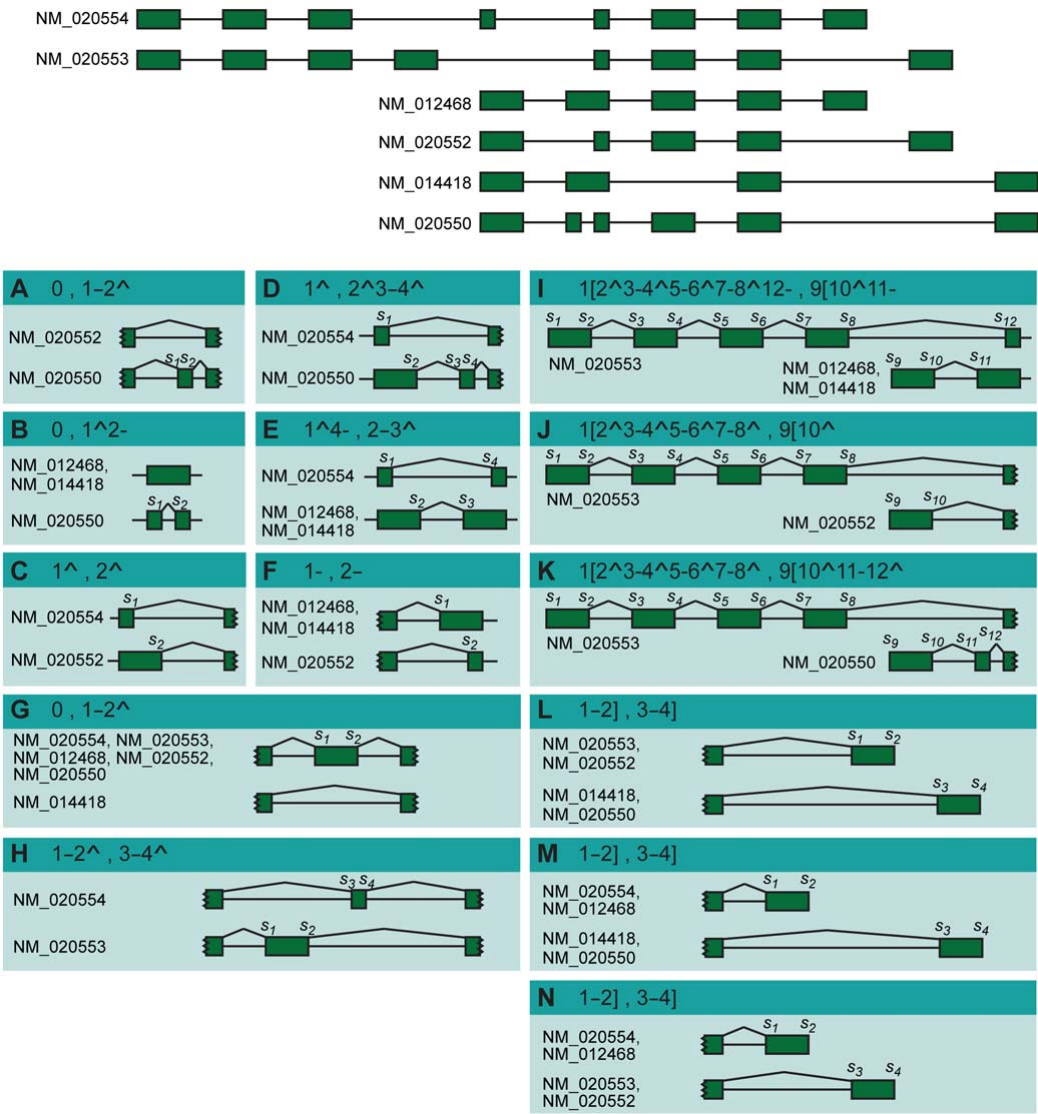
Pairwise AS events in the TCL6 gene. Schematic overview of the RefSeq transcripts of the TCL6 gene (top) and all pairwise AS events (A–N) they describe according to Definition 4. For each event, the corresponding AS code and the structure with the variable splice sites 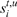 numbered from 5′ to 3′ are presented. Besides traditional events as skipped exon (A and G), retained intron (B), mutually exclusive exons (H), alternative donor (C) and acceptor site (F), novel events are observed that involve more than one of the latter types (D and E) or are connected to differences in the transcription start/polyadenylation site (I through N). Note that in our method L, M and N are considered as three different events that expose the same structure (i.e., [1]–[2],[3]–[4]).

A transcript can be described by a sequence of sites, 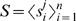 ordered by their genomic positions 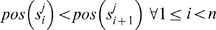. A locus 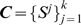 comprises *k*≥1 transcripts that align to a common genomic region (see Materials and Methods, Figure 2). Actually it is reasonable to simultaneously compare the entire set of *k* transcripts from a locus ***C*** when investigating exon–intron variations and AS. However, since it has become popular to compare transcripts in a pairwise fashion, we adapted the subsequent analyzes to the exclusive comparison of transcript pairs {*S^t^*,*S^u^*} ⊆ ***C*** in order to make our results comparable with previous reports. However, we want to stress that pairwise comparisons do not necessarily provide the complete picture of a polymorphic splicing locus, and that the definitions presented in this work can straightforwardly be applied to the comparison of more than two (up to *k*) transcripts in a transcriptional locus ***C***.

#### Definition 2 (Variable Site)

Comparing the exon–intron structure of two transcripts {*S^t^*,*S^u^*}, *variable sites* can be distinguished from sites that are used in both transcripts (“common sites”). A site *s* is said “variable” with respect to {*S^t^*,*S^u^*}, if one and only one of the transcripts exhibits an exon boundary aligning at the genomic position *pos*(*s*), that is |{*S^t^*,*S^u^*} ∩ *transcript*(*s*)| = 1, where |*X*| is the cardinality (the number of elements) of set *X*.

Definition 2 characterizes sites of *S^t^* as variable if they are missing in *S^u^* (and vice versa), regardless whether they map within the genomic region of the primary transcript of *S^u^* or not. Variable sites can thus arise either from alternative transcription initiation (e.g., sites *s*_1_ through *s*_8_ in Figure 2I–K), mRNA cleavage/polyadenylation (sites *s*_3_ and *s*_4_ in Figure 2L–N) or alternative splicing (all other sites in Figure 2). In the latter case, the variable sites should correspond to possibilities for the splicing machinery and we therefore consider a variable splice site as an alternative splice site only if the site is present in the primary RNA sequence of both transcripts, *S^t^* and *S^u^* (Definition 3).

#### Definition 3 (Alternative Splice Site)

Comparing two transcripts 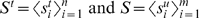, an alternative splice site *s* is a variable site (Definition 2) that (i) is a splice site *type*(*s*) ∈ {*α*,*δ*}, and (ii) is contained within the common genomic region of both transcripts, i.e., 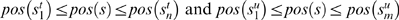.

Alternative splice sites consequently are a subset of variable sites and all splice sites that do not comply with Definition 3 are either used in both transcripts (common sites), or missing in some of them due to alternative TSSs and/or PASs. Note that the same site can be classified differentially with respect to the pair of compared transcripts. For instance, the sites flanking the 4^th^ exon in the transcript NM_020553 are alternative splice sites when comparing with transcript NM_020554 (*s*_1_ and *s*_2_ in Figure 2H) whereas they are variable sites in the comparison with the transcripts NM_012468, NM_014418, NM_020552 and NM_020550 (*s*_7_ and *s*_8_ in Figure 2I–K). Clearly, an AS event should at least contain one alternative splice site. Moreover, as mechanistic interactions between transcription and splicing have been reported [31],[45], variations of transcript initiation/termination have to be included in the AS events occurring at the mRNA extremities. Therefore, we define AS events in a set of different mRNAs as a series of variable sites - with at least one being an alternative splice site—flanked by common sites (Definition 4).

**Definition 4** (AS event): comparing two transcripts (*S^t^*,*S^u^*), an AS event 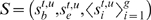 delimited by the common sites 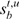 (beginning) and 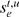 (end) describes a sequence of variables sites 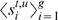 satisfying the following conditions:

- (*consecutiveness of sites*) all sites in *S^t,u^* that are supported by 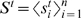 form a consecutive subsequence 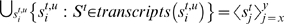 with 1≤*x*<*y*≤*n* (and correspondingly all sites of *S^t,u^* that are in *S^u^*).
- (*minimality of common flanks*) with the exception of the common sites at the flanks of the event 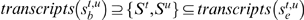, all sites are variable: 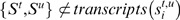 for all 1≤*i*≤*g*.
- (*prerequisite of an alternative splice site*) the variable sites of *S^t,u^* contains an alternative splice site 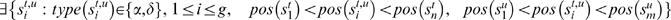

By this, Definition 4 delimits AS events as *g* consecutive variable sites—with at least one alternative splice site—between common sites 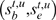 of both transcripts *S^t^* and *S^u^*. In Figure 2, the three first exons of NM_020554 are not involved in an AS with NM_012468 since they are not part of both pre-mRNAs (Definition 4). Note that we create a virtual site upstream and downstream of each locus ***C*** that acts as the first and last site of *all k* transcripts in ***C*** (see Materials and Methods). By this, also AS events that involve alternative TSSs/PASs suffice the criterion of common flanks in Definition 4 (Figure 2).

### A Flexible Code for Alternative Splicing Events

We propose a novel notation system to allow a complete classification of AS events. The general idea is to assign to any AS event a string-based “AS code” that describes the structure of the splicing variation in a concise and univocal manner. AS events of the same type (e.g., exon skipping) are given an identical code and thus can be classified in the same structural group. The codes are built dynamically with respect to each observed splicing variation without the requirement of an a priori defined catalogue of putative AS events. Our notation system is based on the relative position of the variable sites that are involved in the AS event and proceeds as follows: first, all the variable sites of an AS event (see Definition 4) are considered in the order of their genomic position from 5′ to 3′. The indices *i* ∈ N^+^ defined by this relative order are assigned to the corresponding variable sites 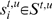. In addition, a symbol is attributed to each site depending on its type. We use the alphabet Σ = {[, ˆ, -,]}, where “[” denotes a TSS 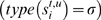, “ ˆ” a splice donor 
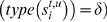
, “-” an acceptor 
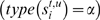
, and “]” a PAS 
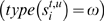
. Therefore, each site is represented by a number (the relative position *i*) and a symbol (identifying the type). To describe one of the splicing structures resulting from an AS event, the number and the symbol of all of the sites that are used by the corresponding mRNA within the event are concatenated into a string. The digit “0” is used if the transcript does not use any variable site (for instance by skipping an exon). The AS code of the event corresponds to the concatenation of these strings, separating the descriptions of the variants by a comma. We order the strings according to the relative position of their first site. Examples are presented in Figures 1 and 2.

Using this notation, AS events with identical codes are *structurally equivalent*, e.g., all exon skipping or all alternative donor events. Moreover, a specific AS code can always be defined for any splicing variation, which guarantees the exhaustiveness of the notation system. For instance, the nonconventional events observed in Figure 1 are assigned the codes (A) 1ˆ3-,2ˆ4-, (B) 1–3ˆ,2–4ˆ, and (C) 1ˆ4–5ˆ6–7ˆ8-,2ˆ3-. Globally, the distribution of AS events into distinct structural classes forms the *landscape* of alternative splicing encompassed in a given annotation.

### Implementation

AStalavista is a JAVA-based tool designed to extract and visualize the structural landscape of AS events as reflected by a given annotation. The input is provided in GTF format, containing the genomic coordinates of exons in the transcripts (and, optionally, the coordinates of the coding regions). AStalavista can be applied to any species for delineating the AS landscape from a whole genome annotation, or to a subset of genes composed according to custom criteria. The output depicts the AS landscape by giving a summary of all pairwise AS events grouped into structurally equal classes which are ranked according to their observed abundances. The web server [42] (http://genome.imim.es/astalavista) has been upgraded and depicts the spectrum of AS structures as described in this manuscript, including variable TSSs/PASs as pointed out by Definition 3 and Definition 4. This means that it is now possible to investigate for instance potential correlations between AS and alternative transcription initiation. Also, the number of species and reference annotations that are supported has been increased.

To assess the agreement of AS events predicted according to our definition with data available from public sources, we compared the output of AStalavista for 5 well studied genes with the events classified for these in recently published or updated databases (Table 1). Since AStalavista is a method rather than a fixed database, the number of AS events that are predicted crucially depends on the transcript annotation(s) under consideration. Therefore, we conducted a first comparison of events extracted by AStalavista from mRNA annotations in Genbank [46] with the EuSplice database that is based on gene annotations. In another run, we enriched the input data by ESTs from dbEST [47] and compared the corresponding results to the EST-based databases ASD, ATD and Hollywood. In order to make the number of events in AStalavista quantitatively comparable with the number of events from public databases, we disregarded in either case AS events predicted in correlation with alternative transcription initiation or polyadenylation. Table 1 shows that AStalavista clearly finds more bona fide events in either dataset than is available from public databases.

**Table 1:** Number of AS events found by AStalavista in comparison to the number of events available from public databases.

Gene	mRNA dataset		EST enriched dataset				
	AStalavista	EuSplice	ASAPII	ASD	AStalavista	ATD	Hollywood
FOXP2	10	3	n/a	n/a	24	n/a	6
DSCR2	1	1	4	6	48	6	3
TTYH1	3	1	8	6	51	4	7
OSCAR	4	5	7	n/a	17	1	n/a
IRAK1	4	2	3	11	80	11	11

In order to allow an objective quantitative comparison, events that incorporate exclusively complete mRNAs (left) have been separated from those that additionally include ESTs (right). For each of the five tested genes (FOXP2—forkhead box 2, DSCR2—down syndrome protein 2, TTYH1—tweety 1 isoform 2, OSCAR—osteoclast-associated receptor, IRAK1—interleukin 1 receptor associated kinase) the number of events is given while “n/a” indicates that a certain gene is not contained in the corresponding database.

We additionally set off to investigate the overlap of the events in a case study (Figure S2) and found that in the FOXP2 gene AStalavista (Figure S2A) finds 5 out of 6 events reported by Hollywood (Figure S2B) and 2 out of 3 events in EuSplice (Figure S2C): in one instance Hollywood marked an alternative splice donor with a very untypical sequence that is supported exclusively by 2 ESTs (Figure_S2B), and in the other case EuSplice predicted a cryptic exon based on the alignment of 2 nt in an intronic stretch which subsequently is tagged with the warning “short exon” and excluded from the analysis on splice site sequences (Figure S2C). For those AStalavista events that are not retrieved from both reference databases (8 out of 10 for EuSplice and 19 out of 24 for Hollywood), we found in total 4 cases that—although the evidence is present in the reference database—have not been reported, probably due to a limitation of the applied classification scheme. These cases are: 0,1–2ˆ3–4ˆ (i.e., the skipping of two consecutive exons in events 14 and 15), 1–2ˆ,3–4ˆ (the mutually exclusive exons in event 23) and 1–2ˆ3-,4- (the skipping of an exon when an alternative downstream acceptor is used, event 24).

### Assessing the Landscape of AS Patterns in Human Reference Annotations

We ran AStalavista on three human popular annotation datasets, namely RefSeq [12], EnsEmbl [48] and Gencode [49]. With our clustering method (see Materials and Methods), the 25,170 RefSeq transcripts clustered into 18,334 loci, the 43,102 EnsEmbl transcripts into 22,303 loci, and the 1,352 coding transcripts of Gencode into 381 loci (Table 2). The differences in the average number of coding transcripts per locus between these annotations (1.4 for RefSeq, 1.9 for EnsEmbl, and 3.6 for Gencode) reflect the differences in exhaustiveness among them. We extracted all variations of the exon–intron structures according to Definition 4. To compensate for artefacts that may occur in automatic annotation pipelines, we omitted AS events that involved introns with no canonical splice site dinucleotides (i.e., not GT/AG). Note that this filtering step consumes a considerable part of the observed running time (Table 2), since for each intron the splice site nucleotides are extracted from the genomic sequence. As expected, the observed running times reflect the number and distribution of transcripts in each input annotation and the longest run (for EnsEmbl) took a bit more than a minute (Table 2).

**Table 2:** Splicing characteristics of different human reference annotations.

	Loci	Transcripts	Exon–intron structure variations	AS event (GT/AG)	Computation time (ms)
Gencode	381	1,352	6,355	548	5,556
RefSeq	18,334	25,170	12,497	4,615	25,638
EnsEmbl	22,303	43,102	59,676	12,206	67,917

Coding transcripts from 3 reference annotations of the human genome—namely, Gencode, RefSeq, and EnsEmbl—have been evaluated for their splicing properties. For each reference annotation, the number of loci obtained by our clustering method in comparison to the number of transcripts is given. Subsequently we present the total number of variations in the exon–intron structure detected by applying the AStalavista method and the subset of them that forms AS events according to Definition 4 with canonical splice sites. Finally, the computation time on a standard desktop PC is reported.

**Figure 3:**
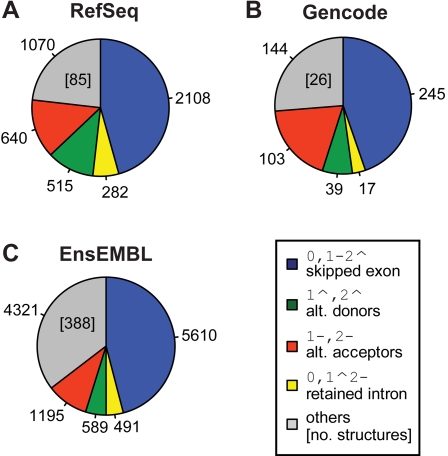
Comparison of the AS landscape in human reference annotations. Distribution of AS events that are not related to alternative transcription starts/polyadenylation sites and contain exclusively introns with canonical splice sites in different reference annotations of the human genome: EnsEmbl, RefSeq, and Gencode. Numbers represent the event count for each different structure and the proportions of the 4 simplest splicing patterns are colored as follows: exon skipping in blue, alternate donors in green, alternate acceptors in red and retained introns in yellow; the fraction of all types of more complex events is shown together in grey with the number of different structures observed there given in brackets. In general, the landscape of AS splicing is similar across the three datasets, with the biggest difference being a comparatively larger fraction of complex events in EnsEmbl.

Next, we analyzed the transcript diversity by characterizing the AS landscapes produced by AStalavista from the different annotations (Figure 3). To compare the results with other studies, we focused on the traditional AS events that present a “simple” splicing pattern—involving at most two alternative splice sites and not correlated with variable TSS/PAS. Agreeing with previously reported observations [29], these simple events are equally ranked from the most abundant to the less in all annotations data sets in the order: exon skipping (ES), alternate donor (AD), alternate acceptors (AA) and intron retention (IR). All other AS events are pooled together (Figure 3, grey sectors in the pie diagrams). These “complex” events form as a whole a substantial part of the AS landscape (from 23.18% in RefSeq up to 35.4% in EnsEmbl), and each of them can be unambiguously described by the notation proposed herein. The composition of these events varies (Table S1): the 1,070 AS events detected in RefSeq correspond to 85 structural distinct classes (A), whereas the 4,321 events in EnsEmbl show 388 classes (C). The fairly most abundant of these complex event (from 25.6% of them in EnsEmbl to 32.6% in Gencode) is the skipping of two exons in a row (0,1–2ˆ3–4ˆ). Mutually exclusive exons (1–2ˆ,3–4ˆ) are less frequent (from 12% to 14.5%), probably due to a more complex molecular mechanism that regulates them. As expected, the higher the complexity of an event—as measured by the number of splice sites involved—the lower its relative abundance. For instance, the “triple exon skipping” (0,1–2ˆ3–4ˆ5–6ˆ) forms ∼7–9% of the complex events. The fact that this event still represents 93 reported cases in the RefSeq annotation (Table S1) illustrates the need for an exhaustive AS notation system and for the corresponding retrieval method.

Obviously, there are differences in the AS landscape between the different reference annotations. This probably reflects the differences in biological data and in the annotation process: manually reviewed full-length cDNA sequences in RefSeq, automatically annotated proteins/cDNAs in EnsEmbl and manually annotated transcripts including ESTs evidence augmented by experimentally verified computational predictions in Gencode. Nevertheless, the different proportions of events agrees with previous results (e.g., [29],[37]) and their ranking is consistent across the sets, which illustrates the general consistence in the AS taxonomies reflected by these annotation systems. Particularly relevant is, in our opinion, the consistency in the AS landscape between the RefSeq and the much richer Gencode annotation. Even though Gencode contains 2.5-fold the number of alternative transcripts per locus, it includes only a marginally larger proportion of the “other” complex AS events than the conservative RefSeq, indicating that while only a fraction of the protein coding transcripts in the human genome may be currently known, the broad AS landscape characterizing the RefSeq annotation is also likely to characterize the entire human transcript complement.

### Differences of the AS Landscapes between 5′ UTR and CDS

**Figure 4:**
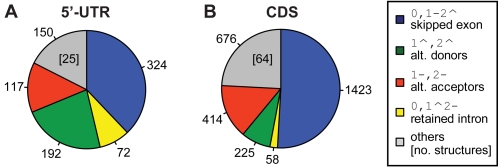
Landscape of AS events in the 5′ UTR vs. CDS. Landscape of AS events in RefSeq with all variable splice sites included in the 5′ UTR (A) in comparison to the ones included in the genomic region of the CDS (B). The structurally different groups are colored as in Figure 3. ES is more frequent in the CDS, whereas IR is observed more often in the 5′ UTR. Whereas in CDS alternative acceptors are more frequent than alternative donors, the landscape of events in the 5′ UTR exhibits a reverse ratio with a bias against alternative acceptors. The more complex AS events are mainly located in the region of the CDS.

We have investigated the differences in the type of AS events occurring in the CDS (coding sequence) from those occurring only in the 5′ UTR (5′ untranslated region). Figure 4 shows the distribution of the simple AS events in 5′ UTRs and in CDSs from the RefSeq annotation. The distribution in 3′ UTRs (3′ untranslated regions) is not shown because of the low frequency of (alternative) splicing in these regions. The analysis here focuses on events completely *included* in a certain region (see Methods)—i.e., in the 5′ UTRs or in the CDSs—but the same trends can be observed for events *overlapping* the 5′ UTR and the CDS (Figure S3). The distributions differ even in the ranking of the four most abundant events. In agreement with [29], the proportion of ES is significantly higher in CDS (50.9% of the landscape) than in the 5′ UTR (37.9%, *p*-value<10^−4^, *χ* ^2^ test). A straightforward explanation is the fact that ES requires at least two introns, which are present in a minority of 5′ UTRs. Coherently with this explanation, we observe the following low proportions of complex AS events in 5′ UTRs vs. CDS: 26.6% vs. 33.1% for 0,1–2ˆ3–4ˆ events (skipping of two exons), 10.6% vs. 17.3% for 1–2ˆ,3–4ˆ events (mutually exclusive exons) and—more drastically—1.3% vs. 10.7% for 0,1–2ˆ3–4ˆ5–6ˆ (the joint skipping of 3 neighboring exons) events. Expectedly, since retained introns in CDSs are likely to introduce in-frame stop codons, the relative proportion of IR is much higher in 5′ UTR (8.4% vs. 2.1%, *p*-value<10^−4^, *χ* ^2^ test). Strikingly, the relative frequency of AA and AD events shows a “reciprocal asymmetry” between the CDS and the 5′ UTRs. In the CDS, the proportion of AAs is nearly twice as high as the proportion of ADs (14.8% vs. 8%), while in the 5′ UTR regions the ratio is the other way around (13.7% vs. 22.5%). Considering findings on the possibly differing molecular mechanism for short range variations at the donor and acceptor site [50]–[52], we repeated the analysis disregarding variations between AD or AA shorter than 5 bp and found a comparable asymmetry (data not shown).

The bias against AAs in 5′ UTRs can be explained by the shorter sequence span where alternate acceptor sites can appear without disrupting the downstream protein sequence. Indeed, if we consider the 5′ UTRs that contain exactly one intron (75% of the spliced 5′ UTRs), the length of the potential target for alternative upstream donor site creation, that is the first exon, is significantly larger than the length of the potential target for alternative downstream acceptor sites creation in 5′ UTR, that is from the acceptor site to the ATG codon (260 vs. 47 nucleotides on average). In order to confirm that the bias against AAs in the 5′ UTR is mainly due to constraints of the start codon, we considered in multi-intronic 5′ UTRs the AS events that do not affect the last intron. Then, the AD/AA ratio drops from factor >1.64 to factor 1.2 (30 AD events compared to 25 AA events in RefSeq). In our opinion, the remaining polarity stems from the fact that the first exon is significantly longer than the second (median 149 vs. 137, p-value ∼3e-6, Kolmogorov-Smirnov-Test), probably resulting from differences in the mechanism for exon definition [27].

**Figure 5:**
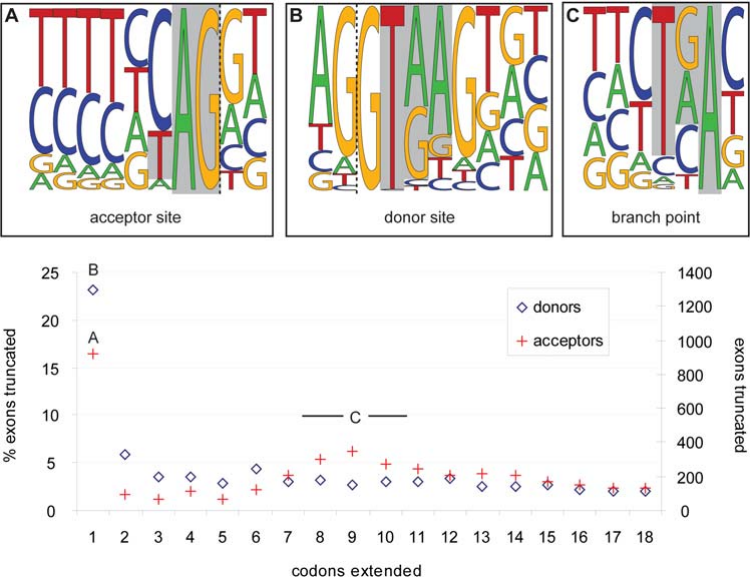
Bias of potential stop codons in the splice site sequences. Proportion of the coding exons that truncate the ORF when artificially extended into the intronic region at the splice donor (blue diamonds) or splice acceptor sites (red crosses). The horizontal axis shows the number of artificial codons taken from the intronic sequence (i.e., the 1^st^, 2^nd^, 3^rd^, etc. codon *downstream* of the splice donor respectively *upstream* of the splice acceptor). The vertical axis to the left gives the percentage of sites that show an in-frame stop with the theoretical inclusion of the respective codon. For the regions A, B, and C, sequence logos are shown where dotted lines indicate the exon boundary and intrinsic potential stop codons are shaded in grey. When regarding exclusively the extension of one (complete) codon into the intron, one third less ORFs would be truncated when extending at the acceptor site compared to the donor site (A vs. B). The observation can partially be explained by in-frame stop codons intrinsic to the different splice site consensus sequences. A secondary peak of stop codons is observed ∼9 extended codons upstream of the acceptor site at a common position for the branch point (consensus sequence C). Sequence logos have been produced with the tool “seqlogo” [66]. Branch point sequences have been kindly provided by the Ast laboratory (http://ast.bioinfo.tau.ac.il/BranchSite.htm).

On the other hand, the observed asymmetry against ADs in the CDS can be explained by the propensity towards the creation of stop codons when considering alternative downstream donor sites, due to the peculiar composition of the donor site consensus sequence. As already reported in the past [53], splicing consensus sequences harbor a high content of intrinsic stop codons (shaded grey in Figure 5A and 5B). To test this hypothesis, we have artificially extended constitutively chosen exon boundaries into the intronic flanks and measured the frequency of in-frame stop codon occurrence separately for the 5′ and the 3′ end. As summarized in Figure 5, the inclusion of one additional codon from the intronic sequence already interrupts the CDS at the donor site ∼50% more often than at the acceptor site. Interestingly, another—though lower—peak of potential stops at the acceptor site is observed after ∼9 codons of extension and coincides with the common location of the branch point consensus (Figure 5C). This difference of potentially included stop codons biases against ADs up to 22 codons of extension (Figure S4) and therefore gives strong evidence for the more frequent use of AAs at flanks of coding exons—albeit more complex mechanisms are also expected to play an additional role.

### AS in Noncoding Transcripts

**Figure 6:**
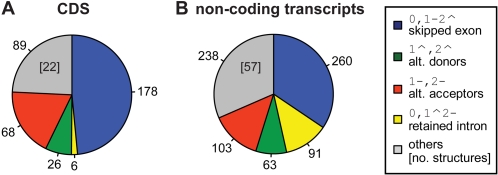
Landscape of AS in noncoding transcripts. The landscape of AS in CDSs of coding transcripts (A) compared to events occurring in noncoding transcripts (B) with the different classes colored as in Figure 3. Complex events and retained introns are more frequent in noncoding transcripts whereas the fraction of ES is clearly higher in coding regions. Alternative donors compared to alternative acceptors are more frequent in the noncoding transcripts.

Additional evidence of the strong effects of the protein coding constraints in shaping the AS landscape comes from the comparison of AS in protein coding and noncoding transcripts. For this comparison, the Gencode annotation is particularly appropriate: it contains many non protein-coding transcripts (2,247 vs. 1,332 coding transcripts), most of them actually occurring also in protein coding loci. In other words, protein coding loci seem to be able to encode both, protein coding and noncoding transcripts. Figure 6 shows the distribution of the AS events in protein coding regions (i.e., in the CDSs) and in noncoding transcripts. The differences are substantial, interestingly also in comparison to the AS events in 5′ UTRs (Figure 4), not biased by the difference in size between the datasets (Figure S5). Almost one third (31.5%) of the AS events observed in noncoding transcripts correspond to complex splice events, compared to only about one fourth (24.3%) in CDSs. Also, the composition of the complex fraction in noncoding transcripts is richer (57 structural different classes vs. 22 in CDSs). Consequently, simple events that are frequently reported in the CDSs of Gencode transcripts are relatively less abundant in noncoding transcripts (e.g., from 48.5% to 34.4% for exon skipping). Naturally, we observe a relaxation of selective constraints against retained introns that make up ∼12% of the landscape in transcripts without an annotated reading frame. The AA/AD ratio is more balanced in noncoding transcripts (1.6 vs. 2.6 in CDSs). The remaining polarity stems from asymmetries in the first compared to the last intron: whereas an alternative TSS in the first exon is often associated with an alternative first donor site (87 instances), an alternative acceptor site in the last exon is less frequently observed with a different PAS (56 cases). When taking into account such events, the numbers for variable 5′ and 3′ flanks of exons are about equal (150 ADs and 159 AAs). This indeed underlines the very different selective constrains acting on coding and noncoding transcripts—even though they may be extensively sharing the same genomic space.

### Distribution of AS Events throughout Metazoan Genomes

**Figure 7:**
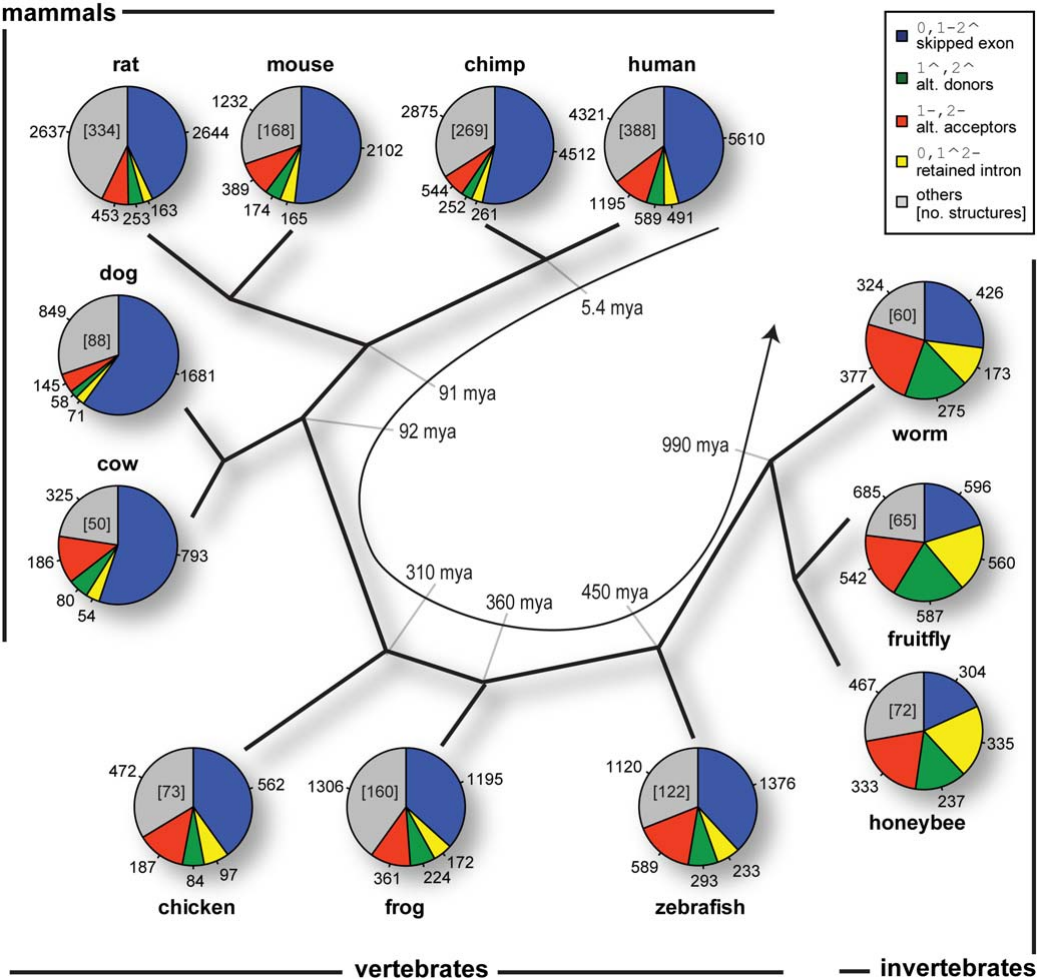
Comparative genomics of the AS landscape in 12 metazoa. For each of the 12 compared species a pie diagram shows the distribution of events across 5 structural different classes (color scheme as in Figure 3). Vertebrates—amongst them especially mammals—exhibit more exon skipping and complex events and less retained introns than invertebrates. Estimations of evolutionary distances are given according to [67].

To investigate the evolution of the AS landscape, we have applied AStalavista to the annotation of 12 different metazoan genomes: human (*Homo sapiens*), chimp (*Pan troglodytes*), mouse (*Mus musculus*), rat (*Rattus norvegicus*), dog (*Canis familiaris*), cow (*Bos taurus*), chicken (*Gallus gallus*), frog (*Xenopus tropicalis*), zebrafish (*Danio rerio*), honeybee (*Apis mellifera*), fruitfly (*Drosophila melanogaster*), and worm (*Caenorhabditis elegans*). While many of the fluctuations observed are likely due to the species-specific differences in amount and quality of the transcriptional data from which the annotations have been derived, our study reveals some interesting trends, suggesting overall that AS patterns did not change gradually but rather abruptly during metazoan evolution (Figure 7). More specifically, IR events are clearly more abundant in invertebrates than in vertebrates. This is consistent with the fact that invertebrates have much shorter introns. Indeed, one could think that IR events involving short introns are less likely to be negatively selected, since the probability for the protein sequence to get disrupted by the introduction of a stop codon is lower than with long introns (Table S2). On the other hand, vertebrates—and especially mammals—exhibit a higher proportion of ES events, while, in contrast, relying relatively less on the usage of alternative donors and acceptors. This may reflect a higher level of regulation of AS in vertebrates, possibly correlated with a higher frequency of exon shuffling and protein domains rearrangements [54]. Finally, we observe an accumulation of complex events in vertebrate genomes compared to the invertebrates (Figure 7). This could be due to the larger number of exons per gene on average in vertebrate genomes (Table S3), which allows to increase the combinatory level, but it also suggest a higher level of sophistication in the control of AS in vertebrate genomes when compared to invertebrates.

## Discussion

Alternative Splicing increases enormously the encoding capacity of the genome of the higher eukaryotic organisms. Its differential regulation is likely to play a substantial role in defining the phenotype of a given cell type, or cell state. We have developed a method to automatically catalogue the patterns of AS events occurring in a given gene/transcript annotation. The method (and the resulting) taxonomy relies on a precise definition of AS event. We have implemented the method in a publicly available software system, named AStalavista.

As a proof of concept, the application of AStalavista to a number of popular annotations of the human genomes has revealed the existence of a plethora of AS types that are usually ignored in published analyses. Indeed, about one quarter of all AS events in these collections belong to this category. Some of these complex AS events, like double exon skipping or mutually exclusive exons, are likely to be under specific regulation. In addition, we report notable differences in the AS landscape between coding and noncoding regions and transcripts, with the landscape in coding regions being largely modelled by protein coding constraints and the landscape in noncoding transcripts suggesting a relaxation of selective constraints.

Our comparison of the AS landscape across 12 metazoan genomes reveals strong differences between vertebrate and non-vertebrate genomes. We observe a higher fraction of intron retention events in invertebrates, while in contrast exon skipping and complex splicing events are more prevalent in vertebrates. While the latter could simply reflect the richer transcript data available for vertebrate, and specifically mammalian genomes, we think that the data is overall suggestive that AS is both more complex and more regulated there, an hypothesis which is compatible with recent studies, according to which there was a substantial increase in AS in the lineage leading to vertebrates, after the separation from invertebrates [55].

Our studies, which we have performed here as a proof of concept of our method, illustrate the potentiality of the AStalavista system to globally characterize the AS landscape of transcriptomes. One could think of many other scenarios—in addition to the basal characterization of the AS landscape in the genome of newly sequenced species—where the characterization of the AS landscape by our system could be of interest. For instance, the AS landscape could be compared across genes clustered in different functional classes, as defined for example by the Gene Ontology project [56], or according to their level or their pattern of expression, or to their conservation across evolution, or to the analyzed tissue or cell type, etc.—in general modulus any biologically relevant partition of the genes from a given species that one can possibly delineate. With the generalization of the new generation of high throughput sequencing instruments, our capacity of effectively surveying various transcriptomes will be greatly enhanced. Differences in such AS landscapes may help to reveal the underlying biological mechanisms responsible for specific phenotypes of the cell (for instance in cancer cells), by pinpointing general splicing de-regulation accidents leading to an alternation of the splicing patterns.

One issue that may remain controversial is the grouping of transcripts into loci, within which the transcripts will be compared in order to identify the occurring AS events. Different groupings may indeed lead to different sets of AS events. Intuitively, one would expect AS to be investigated by comparing transcripts from the same gene. However, recent in-depth annotations projects have had the effect of blurring gene boundaries, up to challenging the definition of a gene [57],[58]. Also, since cases of overlapping transcripts from hitherto distinctly annotated genes are increasingly reported [59],[60], genes can no longer be regarded as isolated units of transcription. Transcription Induced Chimeras [60]–[62], i.e., genes that are fused by a transcript sharing at least one splice site with either one of them, are to be respected when investigating the phenomenon of AS. Therefore, AStalavista includes its own clustering schema in order to ensure an exhaustive detection of AS events, by pooling in a single transcriptional locus all transcripts that overlap on the same strand of the genome sequence. Using these loci instead of the native gene names, we can objectively compare AS classifications across gene sets that involve different criteria for assigning transcripts to genes. In any case, we believe that the introduction of a consistent and rigorous definition of alternative splicing event, which allows in particular a standard characterization of the AS landscape of a given transcriptome, will certainly contribute to a better understanding of the phenomenon of Alternative Splicing.

## Materials and Methods

### Datasets

Annotated transcripts for RefSeq and Gencode (March 2007 freeze) have been downloaded from the UCSC genome browser (http://genome.ucsc.edu) and the annotations for 12 metazoan genomes from EnsEmbl (build 43, http://www.ensembl.org). RefSeq is a nonredundant dataset of gene annotations generated by human supervised alignments of cDNA sequences to the genome [12]. EnsEmbl is a semi-automatic annotation system relying mainly on protein-to-genome sequence alignments [48]. Gencode (http://genome.imim.es/gencode/) is based on the human supervised mapping of all available ESTs, cDNAs and protein sequences onto the Encode regions of the genome [63], which is augmented with computational predictions, and subsequently verified experimentally by RT-PCR and RACE [49]. Additional data in the comparison of metazoan genomes has been obtained from the EnsEmbl web server, containing the version 43 (February 2007) of the EnsEmbl annotation [48] for most of the species, the currently discontinued version 38 (April 2006) of the EnsEmbl annotation for *A. mellifera*, the FlyBase (March 2006) annotation for *D. melanogaster* [64], and the WormBase (May 2006) annotation for *C. elegans* [65].

In each annotation dataset, transcripts that align to genomic regions overlapping on the same strand are clustered into common loci. To avoid some alignment/annotation errors in the datasets, we applied a filtering step discarding all subsequently extracted AS events which contain intron(s) that do not exhibit the consensus dinucleotides GT/AG at their extremities. To assign AS events to a certain region of a gene (e.g., 5′ UTR or CDS), we required that all of the variable sites of the event are located in the respective region. Events spanning more than one region, by this, are excluded in the respective analysis. For the analysis of AS in noncoding transcripts, transcripts with an annotated reading frame have been filtered off the dataset before extracting AS events.

### A Graph Theoretical Approach To Extract Pairwise AS Events

In this section we present the method used in AStalavista to (1) build a splicing graph from a set of transcripts mapped to the genome and (2) efficiently process this graph to extract all pairwise AS events. To infer a splicing graph (see Introduction), the first step is to retrieve the exon boundaries *s_i_* from all transcripts in a locus *C*. To ensure that the sites of a transcript *s_i_* ∈ *S^t^* preserve the usual 5′→3′ directionality in the order given by *pos*(*s_i_*), we artificially invert the genomic coordinates of sites that align to the negative strand. Therefore, splicing graphs *G* = (*V*,*E*) herein are directed acyclic graphs with each node *s* ∈ *V* representing nonredundantly a site of the transcripts in *C*. Each edge (*s_i_*→*s_j_*) ∈ *E* corresponds to an exon (*type*(*s_i_*) ∈ {*α*,*σ*}) or intron (*otherwise*) delimited by *pos*(*s_i_*) and *pos*(*s_j_*) and supported by the transcripts *transcripts*(*s_i_*) ∩ *transcripts*(*s_j_*)≠{}. Note that *G* is non-redundant, i.e., each splice site *s_i_* and each exon/intron (*s_i_*→*s_j_*) is stored once, regardless of the number of transcripts that support it. In order to include AS events associated with variable TSSs and PASs (Definition 4), the graph is completed by the addition of two terminal nodes: a root node *root* (*pos*(*root*) = −∞, *type*(*root*) = Α, *transcripts*(*root*) = *C*) that connects to all TSSs and a leaf node *leaf* (*pos*(*leaf*) = ∞, *type*(*leaf*) = Ω, *transcripts*(*leaf*) = *C*) that connects from all PASs, where Α and Ω are unique types to identify the root/leaf.

#### Definition 5 (Variants)

In G, variants are paths 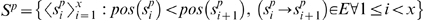 that exhibit a nonempty intersection of transcript evidence 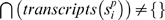. The latter property prevents from connecting freely throughout the graph and creating “hybrid” splicing structures that are not observed in the annotation.

By Definition 5, each variant represents an exonic structure that is supported by at least one transcript evidence.

#### Lemma 1 (Subgraphs Described by Pairwise AS Events)

A pairwise AS event 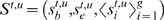 between the transcripts {*S^t^*,*S^u^*} is reflected in *G* by two variants 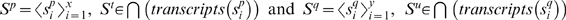 that intersect exactly twice, in their start and end vertices 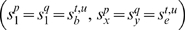.

#### Proof

All sites 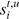 with 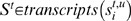 form a variant *S^p^* (condition of consecutiveness in Definition 4) and correspondingly do all sites 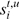 with 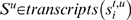. Consequently, the corresponding vertices are connected by edges with at least one common transcript (i.e., *S^t^*, respectively, *S^u^*). The paths *S^p^* and *S^q^* intersect in the common sites flanking the event, 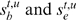 (Definition 4). Furthermore, because of the minimality criterion for common flanks in an AS event, *G* cannot contain any vertex 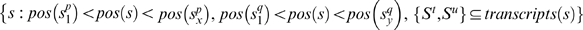.

**Figure 8:**
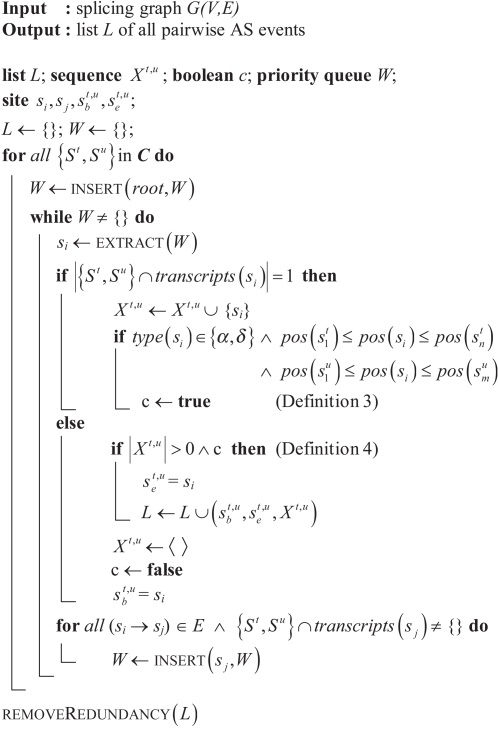
Algorithm for the extraction of pairwise AS events. The algorithm extracts from a splicing graph *G*(*V*,*E*) all events 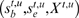 that are described by transcript pairs (*S^t^*,*S^u^*) in a locus *C*. By priority queue *W*, nodes *s_i_* of the splicing graph are iterated from 5′ to 3′ according to *pos*(*s_i_*). The queue contains at the beginning *root* and subsequently is filled with all nodes *s_j_* that are connected by outedges of *s_i_* —if they are supported by either *S^t^* or *S^u^*.

To exhaustively extract pairwise AS events, *G* has to be decomposed into all of the possible subgraphs that suffice Lemma 1. Since the graph structures described in Lemma 1 are necessary but not sufficient for all criteria of Definition 4, *S^p^* and *S^q^* have additionally to be checked for the presence of an alternative splice site. To this end, for each possible transcript pair (*S^t^*,*S^u^*) in a locus ***C***, AS events are retrieved by the iteration sketched in Figure 8.

The algorithm proceeds as follows: In a priority queue *W*, all nodes *s_i_* of *G* that are supported by at least one of the compared transcripts (*S^t^* or *S^u^*) are iterated according to their genomic position *pos*(*s_i_*), from 5′ to 3′ starting with *root* and ending at *leaf*. As by Lemma 1, the algorithm collects successively sequences *X^t,u^* of sites alternatively used in one of the transcripts (|{*S^t^*,*S^u^*} ∩ *transcripts*(*s_i_*)| = 1) flanked by common sites 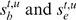 (|{*S^t^*,*S^u^*} ∩ *transcripts*(*s_i_*)| = 2, intrinsic to the else condition since 1≤|{*S^t^*,*S^u^*} ∩ *transcripts*(*s_i_*)|≤2 µ *s_i_* ∈ *W*). In order to suffice Definition 4, these sequences are additionally checked for the presence of an AS site (boolean c) before the event 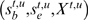 is added to *L*, the list of AS events. Because all transcript pairs (*S^t^*,*S^u^*) in ***C*** are iterated, the main loop of the algorithm in Figure 8 may find multiple instances of AS events that are supported by more than one pair of transcripts. Finally, removeRedundancy() coalesces in *L* such events with equal sequences of sites.

### Complexity Estimation

AStalavista implements the graph-theoretical approach as sketched in the previous section for extraction of pairwise AS events from a given annotation. In this approach initially 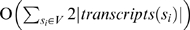 time is required to build up *G* for each locus ***C*** by adding each site annotated in the input to *V* and checking a preceding exonic/intronic edge for eventual creation. Once completely constructed, *G* consumes 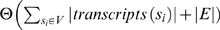 memory.

Making with appropriate data structures the operation {*S^t^*,*S^u^*} ∩ *transcripts*(*s_i_*) feasible in constant time and disregarding the overhead of the operations extract(), respectively, insert() in Figure 8, the time complexity for the extraction of all pairwise events is 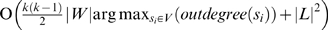, where *k* is the number of transcript variants in ***C***, |*W*| the number of nodes that are supported by one of the transcripts in *S^t^* and/or *S^u^*, *outdegree*(*s_i_*) counting the number of outgoing edges for a node *s_i_* ∈ *V*, and *L* denoting the set of redundant AS events found in ***C***. Obviously, ∼*k* ^2^ pairwise transcript comparisons are to be performed in a locus, for each one the nodes that describe a site of the transcripts are to be iterated and their outedges have to be checked whether they overlap with {*S^t^*,*S^u^*}. Finally, all pairwise events found are to be checked for redundancy in an all-against-all comparison that costs additionally |*L*|^2^. Both quadratic factors, *k* ^2^ and |*L*|^2^, grow naturally with the transcript diversity that is investigated. Reference annotations—even on the complete human genome—are computed in not much more than a minute (Table 2), but the time effort increases when including loci that are annotated extensively with mRNA/EST sequences.

## Supporting Information

Table S1 The landscape of AS in different human reference annotations. Complete landscape of coding transcripts annotated in RefSeq (A), Gencode (B), and EnsEmbl (C). For each different structure, the number of events, their relative abundance (in percent) and the AS code is shown. The 1,070 AS events detected in REFSEQ correspond to 85 structural distinct classes, whereas the 4,321 events in ENSEMBL show 388 classes.
(0.99 MB PDF)

Table S2 Medium exon/intron-length in 12 metazoan species. The EnsEmbl annotations for the genomes of the 12 metazoan species have been used to determine the medium exon and intron length (in nt). Introns with non-canonical splice site dinucleotides (i.e., not GT/AG) and exons that are flanked by such have been disregarded for the analysis. Based on these the median exon and intron length has been estimated, that confirms current estimates: whereas there is not much fluctuation in the median exon length, introns are substantially longer in mammals than in other vertebrates, and even shorter in invertebrates.
(0.10 MB PDF)

Table S3 Attributes of the transcriptome in 12 metazoan species. For each of the 12 species under analysis, this table shows the number of loci (according to the transcript clustering described herein) and the number of transcripts in the corresponding EnsEmbl annotation. Next, the number of variations in the exon-intron structure detected by our method is reported and the subgroup of them that conforms with the requirements for an AS event (Definition 4), exhibits canonical GT/AG splice site dinucleotides and does not involve alternative transcription start/poly-adenylation sites. Finally, the average number of exons per locus that are flanked by canonical GT/AG splice sites is given with the respective standard-deviation across the genome.
(0.24 MB PDF)

Figure S1 UCSC genome browser screenshots for 5 AS events. Screenshots of UCSC genome browser depicting the AS events discussed in Figure 1 in the genes VEGFA (A), CLEC10A (B), TCL6 (C), AURKC (D), and AIF1 (E). Blue boxes are exons, with the coding regions visualized as thicker areas. Chromosomal coordinates and RefSeq identifiers are given to the top respectively to the left.
(0.31 MB PDF)

Figure S2 AS events in the FOXP2 gene. Exploded assembly drawing of the AS events found by AStalavista (A), Hollywood (B), and EuSplice (C) in the FOXP2 gene. The region of events is outlined by a rectangle and double arrows indicate the pairwisely compared variants. The events are numbered consecutively and colors mark different structures: 0,1–2ˆ is blue (events 1–12 and 26), 0,1–2ˆ3–4ˆ is purple (events 13–17), 1-,2- is red (events 18–20), 0,1–2ˆ3–4ˆ5–6ˆ is pink (event 22), 1–2ˆ,3–4ˆ is electric blue (event 23), 1–2ˆ3-,4- is orange (event 24). Hollywood shows splice donor variation (event 25) that is not found by AStalavista since it exhibits the unusual splice donor sequence AAAAT. EuSplice predicts additionally event 26, a cryptic exon that has been inferred from a 2 nt alignment of the mRNA sequence to the genome. In contrast, AStalavista finds 8 more bona fide events with mRNA support than EuSplice and 19 more events in ESTs than Hollywood.
(0.26 MB PDF)

Figure S3 Formed by AS events overlapping the 5′ UTR/CDS. Pie diagrams depicting the landscape of AS events in the RefSeq annotation that are overlapping the respective 5′UTR (A) or the CDS (B) of coding transripts. Qualitatively the same trends can be observed as in Figure 4, events overlapping the CDS show relatively more alternative exons, less alternative introns and much less splice donor variance compared to the acceptor variance.
(0.27 MB PDF)

Figure S4 Cumulative exon truncation at the splice donor/acceptor. The plot shows the cumulative curve for the data presented in Figure 5: hypothetical truncations of the annotated CDSs when extending artificially a certain number of codons (horizontal axis) into the intron from the splice donor (blue diamonds) and acceptor (red crosses) of coding exons. Up to 22 codons of extension, the profile of the splice site sequence causes more exons to be truncated when adopting intronic sequence at the splice donor site.
(0.15 MB PDF)

Figure S5 AS landscape in random subsets of noncoding transcripts. In order to compare the landscape of AS events located in CDSs of coding transcripts (A) with the landscape formed by events in non-coding transcripts (B) in equally sized sets (see Figure 6), 100 datasets of 1,332 noncoding transcripts have been randomly sampled (from the total of 2,247 currently annotated in Gencode) and analyzed. The number of events is presented (arithmetic mean with standard deviation in parenthesis for the 100 random datasets of non-coding transcripts) in structurally different groups (colored according to Figure 3).
(0.30 MB PDF)
