# Supplementary material for: A General Definition and Nomenclature for Alternative Splicing Events
Source: PLoS Comput Biol. 2008 Aug 8;4(8):e1000147. doi: 10.1371/journal.pcbi.1000147 (PMC2467475; doi:10.1371/journal.pcbi.1000147)
Supplement: Table S1 — The landscape of AS in different human reference annotations. Complete landscape of coding transcripts annotated in RefSeq (A), Gencode (B), and EnsEmbl (C). For each different structure, the number of events, their relative abundance (in percent) and the AS code is shown. The 1,070 AS events detected in REFSEQ correspond to 85 structural distinct classes, whereas the 4,321 events in ENSEMBL show 388 classes. (0.99 MB PDF) [file pcbi.1000147.s001.pdf]

A RefSeq

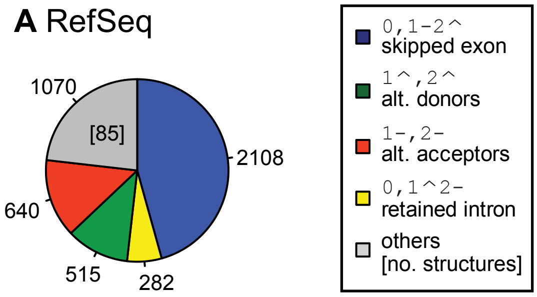

no. events	fraction [%]	AS code
2108	45.68	0, 1-2^
640	13.87	1- , 2-
515	11.16	1^ , 2^
315	6.83	0 , 1-2^3-4^
282	6.11	0 , 1^2-
167	3.62	1-2^ , 3-4^
93	2.02	0 , 1-2^3-4^5-6^
56	1.21	1-2^3- , 4-
53	1.15	1^ , 2^3-4^
44	0.95	0 , 1-2^3-4^5-6^7-8^
37	0.80	1^3-4^ , 2^
30	0.65	1-2^4- , 3-
26	0.56	0 , 1-2^3-4^5-6^7-8^9-10^
22	0.48	1^4- , 2^3-
19	0.41	1-2^ , 3-4^5-6^
16	0.35	1-2^3-4^ , 5-6^
13	0.28	0 , 1^2-3^4-
12	0.26	1-2^3-4^5- , 6-
12	0.26	1^ , 2^3-4^5-6^
10	0.22	1^3- , 2^4-
8	0.17	0 , 1-2^3-4^5-6^7-8^9-10^11-12^13-14^15-16^
7	0.15	0 , 1-2^3-4^5-6^7-8^9-10^11-12^
6	0.13	1- , 2-3^4-
6	0.13	1-3^ , 2-4^
6	0.13	1-2^5-6^ , 3-4^
6	0.13	1-2^3-4^ , 5-6^7-8^
6	0.13	1-2^ , 3-4^5-6^7-8^
5	0.11	1^3-4^5-6^ , 2^
5	0.11	1-2^3-4^5-6^ , 7-8^
4	0.09	1^2-4^ , 3^
4	0.09	1-4^ , 2-3^
3	0.07	1^6- , 2^3-4^5-
3	0.07	1^5-6^ , 2^3-4^
3	0.07	1-2^6- , 3-4^5-
3	0.07	1-2^3-4^6- , 5-
3	0.07	1-2^3-4^5-6^7-8^ , 9-10^
3	0.07	1-2^3-4^5-6^7-8^9-10^11- , 12-
3	0.07	0 , 1-2^3-4^5-6^7-8^9-10^11-12^13-14^15-16^17-18^19-20^
3	0.07	0 , 1-2^3-4^5-6^7-8^9-10^11-12^13-14^15-16^17-18^19-20^21-22^23-24^
2	0.04	1^2-4^5-6^ , 3^
2	0.04	1-2^5- , 3-4^6-
2	0.04	1^3-4^ , 2^5-6^
2	0.04	1^ , 2^3-4^5-6^7-8^

2	0.04	1-2^3-4^5-6^7- , 8-
2	0.04	1-2^3-4^5-6^ , 7-8^9-10^
2	0.04	1-2^3-4^5-6^7-8^9-10^ , 11-12^13-14^
2	0.04	0 , 1-2^3-4^5-6^7-8^9-10^11-12^13-14^15-16^17-18^
1	0.02	1^2- , 3^4-
1	0.02	1^2-3^ , 4^
1	0.02	1-2^3-5^6- , 4-
1	0.02	1-3^6- , 2-4^5-
1	0.02	1^2-4^ , 3^5-6^
1	0.02	1-2^4-5^6- , 3-
1	0.02	0 , 1^2-3^4-5^6-
1	0.02	1-2^5-6^8- , 3-4^7-
1	0.02	1-2^3-4^5-6^8- , 7-
1	0.02	1-2^3-4^5- , 6-7^8-
1	0.02	1-4^5-6^7- , 2-3^8-
1	0.02	1-2^8- , 3-4^5-6^7-
1	0.02	1-2^3-4^8- , 5-6^7-
1	0.02	1-2^7- , 3-4^5-6^8-
1	0.02	1^3-4^5-6^7- , 2^8-
1	0.02	1-2^5-6^7-8^ , 3-4^
1	0.02	1-2^ , 3-4^5-6^7-8^9-10^
1	0.02	1-2^8-9^10- , 3-4^5-6^7-
1	0.02	1^ , 2^3-4^5-6^7-8^9-10^
1	0.02	1-2^5-6^7-8^9-10^ , 3-4^
1	0.02	1-2^3-4^5-6^7-8^9- , 10-
1	0.02	1-2^3-4^ , 5-6^7-8^9-10^
1	0.02	1^ , 2^3-4^5-6^7-8^9-10^11-12^
1	0.02	1-2^3-4^5-6^7-8^ , 9-10^11-12^
1	0.02	1-2^ , 3-4^5-6^7-8^9-10^11-12^13-14^
1	0.02	1-2^3-4^5-6^7-8^ , 9-10^11-12^13-14^
1	0.02	1-2^3-4^5-6^7-8^9-10^11-12^ , 13-14^
1	0.02	0 , 1-2^3-4^5-6^7-8^9-10^11-12^13-14^
1	0.02	1-2^5-6^7-8^9-10^11-12^13-14^15-16^ , 3-4^
1	0.02	1-2^3-4^ , 5-6^7-8^9-10^11-12^13-14^15-16^
1	0.02	1^3-4^5-6^7-8^9-10^11-12^13-14^15-16^17-18^ , 2^
1	0.02	1-2^3-4^5-6^7-8^9-10^11-12^13-14^15-16^ , 17-18^
1	0.02	1-2^5-6^7-8^9-10^11-12^ , 3-4^13-14^15-16^17-18^
1	0.02	1-2^3-4^5-6^7-8^9-10^11-12^13-14^15-16^17- , 18-
1	0.02	1-2^3-4^7-8^9-10^11-12^13-14^15-16^ , 5-6^17-18^19-20^21-22^
1	0.02	0 , 1-2^3-4^5-6^7-8^9-10^11-12^13-14^15-16^17-18^19-20^21-22^
1	0.02	1-2^3-4^5-6^7-8^9-10^11-12^13-14^15-16^17-18^19-20^21-22^23-24^25-26^27- , 28-
1	0.02	1-2^3-4^5-6^7-8^9-10^11-12^13-14^15-16^17-18^19-20^21-22^23-24^25-26^27-28^29- , 30-
1	0.02	0 , 1-2^3-4^5-6^7-8^9-10^11-12^13-14^15-16^17-18^19-20^21-22^23-24^25-26^27-28^29-30^
1	0.02	0 , 1-2^3-4^5-6^7-8^9-10^11-12^13-14^15-16^17-18^19-20^21-22^23-24^25-26^27-28^29-30^31-32^33-34^35-36^
1	0.02	0 , 1-2^3-4^5-6^7-8^9-10^11-12^13-14^15-16^17-18^19-20^21-22^23-24^25-26^27-28^29-30^31-32^33-34^35-36^37-38^

B Gencode

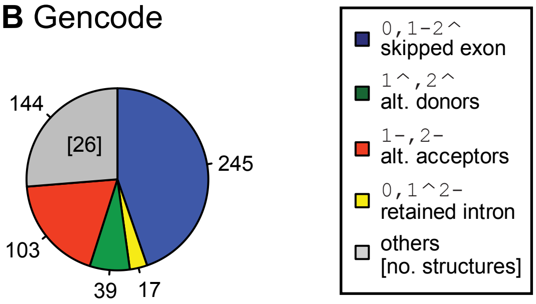

no. events	fraction [%]	AS code
245	44.71	1-2^ , 0
103	18.80	1- , 2-
47	8.58	1-2^3-4^ , 0
39	7.12	1^ , 2^
21	3.83	1-2^ , 3-4^
17	3.10	1^2- , 0
16	2.92	1-2^3- , 4-
10	1.82	1-2^3-4^5-6^ , 0
7	1.28	1-2^4- , 3-
4	0.73	1^3-4^ , 2^
4	0.73	1^3- , 2^4-
4	0.73	1-2^ , 3-4^5-6^
3	0.55	1-2^3-4^ , 5-6^
3	0.55	1-2^6- , 3-4^5-
3	0.55	1-2^3-4^5-6^7-8^ , 0
2	0.36	1^ , 2^3-4^
2	0.36	1-2^3-4^5- , 6-
2	0.36	1-2^5-6^ , 3-4^
2	0.36	1-2^3-4^5-6^ , 7-8^
2	0.36	1-2^3-4^ , 5-6^7-8^
2	0.36	1-2^ , 3-4^5-6^7-8^
2	0.36	1-2^3-4^5-6^7-8^9-10^ , 0
1	0.18	1-3^ , 2-4^
1	0.18	1^2-3^4- , 0
1	0.18	1^3-4^5-6^ , 2^
1	0.18	1-2^8- , 3-4^5-6^7-
1	0.18	1-2^3-4^5-6^7- , 8-
1	0.18	1-2^3-4^ , 5-6^7-8^9-10^
1	0.18	1-2^3-4^5-6^7-8^9-10^11-12^ , 0
1	0.18	1^ , 2^3-4^5-6^7-8^9-10^11-12^13-14^

C EnsEmbl

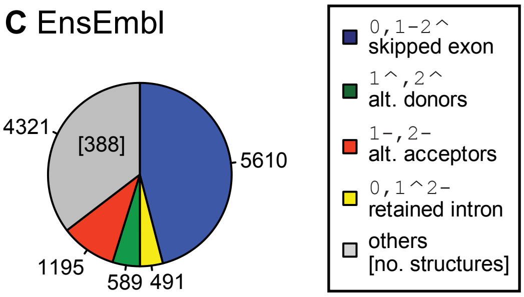

no. events	fraction [%]	AS code
5610	45.96	0, 1-2^
1195	9.79	1- , 2-
1106	9.06	0 , 1-2^3-4^
589	4.83	1^ , 2^
554	4.54	1-2^ , 3-4^
491	4.02	0 , 1^2-
325	2.66	0 , 1-2^3-4^5-6^
171	1.40	1^3- , 2^4-
141	1.16	0 , 1-2^3-4^5-6^7-8^
139	1.14	1-2^4- , 3-
137	1.12	1^3-4^ , 2^
129	1.06	1-2^3- , 4-
112	0.92	1^ , 2^3-4^
77	0.63	1-2^ , 3-4^5-6^
76	0.62	0 , 1^2-3^4-
74	0.61	1-2^3-4^ , 5-6^
57	0.47	0 , 1-2^3-4^5-6^7-8^9-10^
55	0.45	1^4- , 2^3-
43	0.35	0 , 1-2^3-4^5-6^7-8^9-10^11-12^
38	0.31	1-2^5-6^ , 3-4^
37	0.30	1-2^3-4^5- , 6-
33	0.27	1^5-6^ , 2^3-4^
29	0.24	1^ , 2^3-4^5-6^
26	0.21	1^3-4^ , 2^5-6^
26	0.21	1^3-4^5-6^ , 2^
25	0.20	1^6- , 2^3-4^5-
25	0.20	1-2^3-4^6- , 5-
20	0.16	1^8- , 2^3-4^5-6^7-
19	0.16	1-2^6- , 3-4^5-
19	0.16	1^3-4^6- , 2^5-
19	0.16	1-2^3-4^ , 5-6^7-8^
18	0.15	1-2^5- , 3-4^6-
18	0.15	0 , 1-2^3-4^5-6^7-8^9-10^11-12^13-14^
18	0.15	0 , 1-2^3-4^5-6^7-8^9-10^11-12^13-14^15-16^
17	0.14	1-2^ , 3-4^5-6^7-8^
15	0.12	1^5- , 2^3-4^6-
12	0.10	1- , 2-3^4-
12	0.10	1^2-3^ , 4^
12	0.10	1^3-4^5- , 2^6-
12	0.10	1-2^3-4^5-6^7- , 8-
12	0.10	1-2^3-4^5-6^ , 7-8^
11	0.09	1^10- , 2^3-4^5-6^7-8^9-
10	0.08	1^ , 2^3-4^5-6^7-8^
10	0.08	0 , 1-2^3-4^5-6^7-8^9-10^11-12^13-14^15-16^17-18^

10	0.08	0 , 1-2^3-4^5-6^7-8^9-10^11-12^13-14^15-16^17-18^19-20^21-22^23-24^
9	0.07	1-3^ , 2-4^
9	0.07	1-2^3-4^7- , 5-6^8-
9	0.07	1^ , 2^3-4^5-6^7-8^9-10^
8	0.07	1-4^ , 2-3^
8	0.07	1^3-4^5-6^7-8^ , 2^
8	0.07	1^3-4^ , 2^5-6^7-8^
8	0.07	1-2^3-4^5-6^ , 7-8^9-10^11-12^
7	0.06	1^2-4^ , 3^
7	0.06	0 , 1^2-3^4-5^6-7^8-
7	0.06	1-2^3-4^5-6^7-8^9- , 10-
6	0.05	1-2^3-4^7-8^ , 5-6^
6	0.05	1^5-6^8- , 2^3-4^7-
6	0.05	1-2^ , 3-4^5-6^7-8^9-10^
5	0.04	1-3^4- , 2-
5	0.04	1-3^5- , 2-4^6-
5	0.04	0 , 1^2-3^4-5^6-
5	0.04	1^3-4^7- , 2^5-6^8-
5	0.04	1^3-4^5-6^7- , 2^8-
5	0.04	1-2^5-6^8- , 3-4^7-
5	0.04	1-2^3-4^5-6^8- , 7-
5	0.04	1^12- , 2^3-4^5-6^7-8^9-10^11-
5	0.04	1^16- , 2^3-4^5-6^7-8^9-10^11-12^13-14^15-
5	0.04	1^20- , 2^3-4^5-6^7-8^9-10^11-12^13-14^15-16^17-18^19-
4	0.03	1^2- , 3^4-
4	0.03	1^2- , 3-4^
4	0.03	0 , 1-2^3^4-
4	0.03	1-3^6- , 2-4^5-
4	0.03	1-2^4-5^ , 3-6^
4	0.03	1^2-3^6- , 4^5-
4	0.03	1-4^6- , 2-3^5-
4	0.03	1^4-5^6- , 2^3-
4	0.03	1^5-6^ , 2^3-4^7-8^
4	0.03	1^6-7^8- , 2^3-4^5-
4	0.03	1^7-8^ , 2^3-4^5-6^
4	0.03	1-2^7-8^ , 3-4^5-6^
4	0.03	1-2^3-4^8- , 5-6^7-
4	0.03	1^5-6^7-8^9-10^ , 2^3-4^
4	0.03	1-2^3-4^5-6^7-8^9-10^11-12^13- , 14-
4	0.03	1^14- , 2^3-4^5-6^7-8^9-10^11-12^13-
4	0.03	0 , 1-2^3-4^5-6^7-8^9-10^11-12^13-14^15-16^17-18^19-20^
3	0.02	1-2^ , 3^4-
3	0.02	1-2^3-6^ , 4-5^
3	0.02	1^3-4^5-6^ , 2^7-8^
3	0.02	1^3-4^5-6^8- , 2^7-
3	0.02	1^3-4^8- , 2^5-6^7-
3	0.02	1-2^5-6^7-8^ , 3-4^
3	0.02	1-2^8- , 3-4^5-6^7-
3	0.02	1^5-6^7-8^ , 2^3-4^
3	0.02	1^5-6^10- , 2^3-4^7-8^9-
3	0.02	1-2^3-4^5-6^7-8^ , 9-10^
3	0.02	1^9-10^ , 2^3-4^5-6^7-8^
3	0.02	1-2^3-4^5-6^7-8^9-10^11- , 12-
3	0.02	1-2^ , 3-4^5-6^7-8^9-10^11-12^

3	0.02	1^3-4^5-6^7-8^9-10^11- , 2^12-
3	0.02	1-2^12- , 3-4^5-6^7-8^9-10^11-
3	0.02	1^3-4^5-6^7-8^9-10^11-12^13- , 2^14-
3	0.02	1-2^3-4^5-6^7-8^9-10^11-12^13-14^15-16^17- , 18-
3	0.02	0 , 1-2^3-4^5-6^7-8^9-10^11-12^13-14^15-16^17-18^19-20^21-22^23-24^25-26^
3	0.02	0 , 1-2^3-4^5-6^7-8^9-10^11-12^13-14^15-16^17-18^19-20^21-22^23-24^25-26^27-28^
3	0.02	1^30- , 2^3-4^5-6^7-8^9-10^11-12^13-14^15-16^17-18^19-20^21-22^23-24^25-26^27-28^29-
2	0.02	1^ , 2^3^4-5-6^
2	0.02	1^3^4- , 2^
2	0.02	1^ , 2^3^4-
2	0.02	1^2-3^5-6^ , 4^
2	0.02	1-4^5-6^ , 2-3^
2	0.02	1-4^5- , 2-3^6-
2	0.02	1^2-3^ , 4^5-6^
2	0.02	1^2-3^5- , 4^6-
2	0.02	1-2^4-6^ , 3-5^
2	0.02	1^5- , 2^3^4-6-
2	0.02	1^ , 2^3^4-5-6^
2	0.02	1-3^ , 2-4^5-6^
2	0.02	1^3^4-6- , 2^5-
2	0.02	1-2^3-4^6-8^ , 5-7^
2	0.02	1^5-6^7- , 2^3-4^8-
2	0.02	1-2^7- , 3-4^5-6^8-
2	0.02	1^7- , 2^3-4^5-6^8-
2	0.02	1^3-4^7-8^ , 2^5-6^
2	0.02	1^2-3^8- , 4^5-6^7-
2	0.02	1-4^8- , 2-3^5-6^7-
2	0.02	1-2^9- , 3-4^5-6^7-8^10-
2	0.02	1-2^3-4^5-6^9- , 7-8^10-
2	0.02	1-2^3-4^5-6^9-10^ , 7-8^
2	0.02	1-2^8-9^10- , 3-4^5-6^7-
2	0.02	1-2^4-5^7-8^10- , 3-6^9-
2	0.02	1^7-8^ , 2^3-4^5-6^9-10^
2	0.02	1^3-4^6-8^10- , 2^5-7^9-
2	0.02	1^3-4^5-6^7-8^ , 2^9-10^
2	0.02	1-4^5-6^7-8^9-10^ , 2-3^
2	0.02	1^3-4^10- , 2^5-6^7-8^9-
2	0.02	0 , 1^2-3^4-5^6-7^8-9^10-
2	0.02	1^ , 2^3-4^5-6^7-8^9-10^11-12^
2	0.02	1^10-11^ , 2^3-4^5-6^7-8^9-12^
2	0.02	1^5-6^7-8^9-10^11- , 2^3-4^12-
2	0.02	1-2^5-6^7-8^9-10^11- , 3-4^12-
2	0.02	1-3^12- , 2-4^5-6^7-8^9-10^11-
2	0.02	0 , 1^2-3^4-5^6-7^8-9^10-11^12-
2	0.02	1^2-3^ , 4^5-6^7-8^9-10^11-12^13-14^
2	0.02	1-2^14- , 3-4^5-6^7-8^9-10^11-12^13-
2	0.02	1^3-4^5-6^7-8^9-10^11-12^13-14^ , 2^
2	0.02	1-2^16- , 3-4^5-6^7-8^9-10^11-12^13-14^15-
2	0.02	1-2^3-4^16- , 5-6^7-8^9-10^11-12^13-14^15-
2	0.02	1^ , 2^3-4^5-6^7-8^9-10^11-12^13-14^15-16^17-18^
2	0.02	1^10^11-20- , 2^3-4^5-6^7-8^9-12^13-14^15-16^17-18^19-
2	0.02	1-2^3-4^5-6^7-8^9-10^11-12^13-14^15-16^17-18^ , 19-20^
2	0.02	1^17-18^20- , 2^3-4^5-6^7-8^9-10^11-12^13-14^15-16^19-

2	0.02	1-2^3-4^5-6^7-8^9-10^11-12^13-14^15-16^17-18^19-20^ , 21-22^
2	0.02	1^3-4^20-21^ , 2^5-6^7-8^9-10^11-12^13-14^15-16^17-18^19-22^
2	0.02	0 , 1-2^3-4^5-6^7-8^9-10^11-12^13-14^15-16^17-18^19-20^21-22^
2	0.02	1-2^3-4^5-6^7-8^9-10^11-12^13-14^15-16^17-18^19-20^21-22^ , 23-24^
2	0.02	1^24- , 2^3-4^5-6^7-8^9-10^11-12^13-14^15-16^17-18^19-20^21-22^23-
2	0.02	1^15-16^26- , 2^3-4^5-6^7-8^9-10^11-12^13-14^17-18^19-20^21-22^23-24^25-
2	0.02	1-2^3-4^5-6^7-8^9-10^11-12^13-14^15-16^17-18^19-20^21-22^23-24^25-26^27-28^29- , 30-
2	0.02	1^23-24^30- , 2^3-4^5-6^7-8^9-10^11-12^13-14^15-16^17-18^19-20^21-22^25-26^27-28^29-
2	0.02	0 , 1-2^3-4^5-6^7-8^9-10^11-12^13-14^15-16^17-18^19-20^21-22^23-24^25-26^27-28^29-30^31-32^
2	0.02	1^20-21^34- , 2^3-4^5-6^7-8^9-10^11-12^13-14^15-16^17-18^19-22^23-24^25-26^27-28^29-30^31-32^33-
2	0.02	0 , 1-2^3-4^5-6^7-8^9-10^11-12^13-14^15-16^17-18^19-20^21-22^23-24^25-26^27-28^29-30^31-32^33-34^35-36^
2	0.02	1^12-13^44- , 2^3-4^5-6^7-8^9-10^11-14^15-16^17-18^19-20^21-22^23-24^25-26^27-28^29-30^31-32^33-34^35-36^37-38^39-40^41-42^43-
2	0.02	0 , 1-2^3-4^5-6^7-8^9-10^11-12^13-14^15-16^17-18^19-20^21-22^23-24^25-26^27-28^29-30^31-32^33-34^35-36^37-38^39-40^41-42^43-44^
1	0.01	1-3^5-6^ , 2-4^
1	0.01	1-2^ , 3^
1	0.01	0 , 1^2-3^
1	0.01	1^2-4- , 3-
1	0.01	0 , 1^2^3-4-
1	0.01	1-2^3^4-6- , 5-
1	0.01	1-2^3^4- , 5-6^
1	0.01	1^4-5^ , 2^3-6^
1	0.01	1- , 2-3^4-5^6-
1	0.01	1-2^6- , 3^4-5-
1	0.01	1^ , 2^3-4^5^6-
1	0.01	1-2^3- , 4-5^6-
1	0.01	1^3- , 2^4-5^6-
1	0.01	1-3^5-6^ , 2-4^
1	0.01	1-2^3-4^ , 5^6-
1	0.01	1-2^3-5^6- , 4-
1	0.01	1-2^3-5^ , 4-6^
1	0.01	1^3-4^ , 2^5^6-
1	0.01	1^5-6^ , 2^3^4-
1	0.01	1-2^5- , 3^4-6-
1	0.01	1-2^4-5^6- , 3-
1	0.01	1-4^ , 2-3^5-6^
1	0.01	1^3^4-5- , 2^6-
1	0.01	1-2^3-4^5- , 6-7^8-
1	0.01	1-2^3-4^5-6^ , 7^8-
1	0.01	1^4-5^ , 2^3-6^7-8^
1	0.01	1-4^5-6^7- , 2-3^8-
1	0.01	1-2^3-4^7- , 5^6-8-
1	0.01	1-3^7-8^ , 2-4^5-6^
1	0.01	1-2^5-6^7- , 3-4^8-
1	0.01	1^3-6^8- , 2^4-5^7-
1	0.01	1-3^5-6^7-8^ , 2-4^
1	0.01	1^6-8^ , 2^3-4^5-7^
1	0.01	1^5-6^8- , 2^3^4-7-

1	0.01	1-2^4-5^6-8^ , 3-7^
1	0.01	1^5-7^ , 2^3-4^6-8^
1	0.01	1-6^7-8^ , 2-3^4-5^
1	0.01	1-2^5-7^ , 3-4^6-8^
1	0.01	1^3-5^7- , 2^4-6^8-
1	0.01	1-2^3-4^5-8^ , 6-7^
1	0.01	1-4^ , 2-3^5-6^7-8^
1	0.01	1^2^3-4-5^7-8^ , 6^
1	0.01	1-2^3-5^7-8^ , 4-6^
1	0.01	1^7-8^ , 2^3-4^5^6-
1	0.01	1^ , 2^3^4-5-6^7-8^
1	0.01	1^3-4^5-7^8- , 2^6-
1	0.01	1-2^5-6^10- , 3-4^7-8^9-
1	0.01	1^2-5-6^7-8^9-10^ , 3-4^
1	0.01	1-2^3-4^9-10^ , 5-6^7-8^
1	0.01	1-4^5-6^7-8^9- , 2-3^10-
1	0.01	1^7-8^9-10^ , 2^3-4^5-6^
1	0.01	1-2^3-4^5-6^10- , 7-8^9-
1	0.01	1-3^4-5^6-7^8-10^ , 2-9^
1	0.01	1^3-4^5-6^9- , 2^7^8-10-
1	0.01	1^7-8^10- , 2^3-4^5-6^9-
1	0.01	1^3-4^5-6^ , 2^7-8^9-10^
1	0.01	1^8-9^10- , 2^3-4^5-6^7-
1	0.01	1^3-4^ , 2^5-6^7-8^9-10^
1	0.01	1^5-6^7- , 2^3-4^8-9^10-
1	0.01	1-2^3-4^8-9^10- , 5-6^7-
1	0.01	1-2^3-4^5-8^9-10^ , 6-7^
1	0.01	1^3-4^5-6^7-8^9- , 2^10-
1	0.01	1-2^3-4^ , 5-6^7-8^9-10^
1	0.01	1^2-3^5-6^7-8^9- , 4^10-
1	0.01	1-2^3-4^5-6^7-8^10- , 9-
1	0.01	1^3-4^5-7^9-10^ , 2^6-8^
1	0.01	1^3-4^5-6^7-8^9-10^ , 2^
1	0.01	1-2^3-4^7-8^9- , 5-6^10-
1	0.01	1^7^8-10- , 2^3-4^5-6^9-
1	0.01	1^3-6^9-10^ , 2^4-5^7-8^
1	0.01	1^8^9-10- , 2^3-4^5-6^7-
1	0.01	1-2^3-4^5-6^7-8^ , 9^10-
1	0.01	1-2^3-4^10- , 5-6^7-8^9-
1	0.01	0 , 1-2^3-4^5-6^7-8^9^10-
1	0.01	1-2^3-4^12- , 5-6^7-8^9-10^11-
1	0.01	1-2^9-10^11-12^ , 3-4^5-6^7-8^
1	0.01	1^2-4^5-6^7-8^9-10^12- , 3^11-
1	0.01	1^7-8^9-10^ , 2^3-4^5-6^11-12^
1	0.01	1^5-6^7-8^9-10^12- , 2^3-4^11-
1	0.01	1^9^10-12- , 2^3-4^5-6^7-8^11-
1	0.01	1-4^5-6^ , 2-3^7-8^9-10^11-12^
1	0.01	1-2^7-8^9- , 3-4^5-6^10-11^12-
1	0.01	1^3-4^5-6^7-8^9-10^11-12^ , 2^
1	0.01	1^6-8^ , 2^3-4^5-7^9-10^11-12^
1	0.01	1-3^7-8^9-10^11-12^ , 2-4^5-6^
1	0.01	1-2^3-4^ , 5-6^7-8^9-10^11-12^
1	0.01	1^9-10^11- , 2^3-4^5-6^7-8^12-
1	0.01	1-2^7-8^9-10^11-12^ , 3-4^5-6^

1	0.01	1-2^3-4^5-6^7-8^9-10^ , 11-12^
1	0.01	1^2^3-12- , 4^5-6^7-8^9-10^11-
1	0.01	1^6-7^9-10^11- , 2^3-4^5-8^12-
1	0.01	1-2^3-4^5-6^9-10^11-12^ , 7-8^
1	0.01	1^3-4^5-6^8-10^12- , 2^7-9^11-
1	0.01	1^11-12^ , 2^3-4^5-6^7-8^9-10^
1	0.01	1-2^3-4^5-6^7-8^ , 9-10^11-12^
1	0.01	1-2^3-4^10-11^12- , 5-6^7-8^9-
1	0.01	1^7-8^11-12^ , 2^3-4^5-6^9-10^
1	0.01	1^2-3^ , 4^5-6^7-8^9-10^11-12^
1	0.01	1^9-10^12- , 2^3-4^5-6^7-8^11-
1	0.01	1-2^3^4-12- , 5-6^7-8^9-10^11-
1	0.01	1^7-8^ , 2^3-4^5-6^9-10^11-12^
1	0.01	1-2^3-4^5-6^12- , 7-8^9-10^11-
1	0.01	0 , 1-2^3^4-5-6^7-8^9-10^11-12^
1	0.01	1-2^7-8^9-10^11-12^13- , 3-4^5-6^14-
1	0.01	1^13- , 2^3-4^5-6^7-8^9-10^11-12^14-
1	0.01	1^3^4-6-7^9-10^11-12^ , 2^5-8^13-14^
1	0.01	1-2^7-8^9-10^11-12^13-14^ , 3-4^5-6^
1	0.01	1^7-8^11-12^13-14^ , 2^3-4^5-6^9-10^
1	0.01	1-2^3-4^5-6^7-8^14- , 9-10^11-12^13-
1	0.01	1^5-6^8-9^10-12^14- , 2^3-4^7-11^13-
1	0.01	1-2^ , 3-4^5-6^7-8^9-10^11-12^13-14^
1	0.01	1-2^3-4^5-6^14- , 7-8^9-10^11-12^13-
1	0.01	1-2^3-4^5-6^7-8^9-10^14- , 11-12^13-
1	0.01	1-2^3-4^5-6^13-14^ , 7-8^9-10^11-12^
1	0.01	1^ , 2^3-4^5-6^7-8^9-10^11-12^13-14^
1	0.01	1^11-12^14- , 2^3-4^5-6^7-8^9-10^13-
1	0.01	1^3-4^10-11^13-14^ , 2^5-6^7-8^9-12^
1	0.01	1-2^13-14^ , 3-4^5-6^7-8^9-10^11-12^
1	0.01	1-2^3-4^5-6^7-8^ , 9-10^11-12^13-14^
1	0.01	1^7-8^9-10^11-12^13- , 2^3-4^5-6^14-
1	0.01	1-2^5-6^7-8^9-10^11-12^13- , 3-4^14-
1	0.01	1^7-8^9-10^11-12^ , 2^3-4^5-6^13-14^
1	0.01	1^2-3^5-6^7-8^9-10^11-12^13-14^ , 4^
1	0.01	1-2^3-4^14- , 5-6^7-8^9-10^11-12^13-
1	0.01	1-2^5-6^7-8^9-10^11-12^14- , 3-4^13-
1	0.01	1-2^3-4^ , 5-6^7-8^9-10^11-12^13-14^
1	0.01	1-2^3-5^9-10^11-12^13- , 4-6^7-8^14-
1	0.01	1^7-8^14- , 2^3-4^5-6^9-10^11-12^13-
1	0.01	1^7-8^9-10^ , 2^3-4^5-6^11-12^13-14^
1	0.01	1^3-4^5-6^7-8^9-10^11-12^13-14^15-16^ , 2^
1	0.01	1-2^3-4^5-6^7-8^9-10^11-12^13-14^15- , 16-
1	0.01	1^5-6^16- , 2^3-4^7-8^9-10^11-12^13-14^15-
1	0.01	1^7-8^9-10^11-12^ , 2^3-4^5-6^13-14^15-16^
1	0.01	1^3-4^ , 2^5-6^7-8^9-10^11-12^13-14^15-16^
1	0.01	1-2^3-4^5-6^7-8^9-10^11-12^13-14^ , 15-16^
1	0.01	1-2^3-4^5-6^7-8^13- , 9-10^11-12^14-15^16-
1	0.01	1-2^3-4^5-6^7-8^ , 9-10^11-12^13-14^15-16^
1	0.01	1-2^9-10^11-12^13-14^15- , 3-4^5-6^7-8^16-
1	0.01	1-2^3-4^5-6^7-8^16- , 9-10^11-12^13-14^15-
1	0.01	1-2^7-8^16- , 3-4^5-6^9-10^11-12^13-14^15-
1	0.01	1^15-16^ , 2^3-4^5-6^7-8^9-10^11-12^13-14^
1	0.01	1^14-15^ , 2^3-4^5-6^7-8^9-10^11-12^13-16^

1	0.01	1-2^ , 3-4^5-6^7-8^9-10^11-12^13-14^15-16^
1	0.01	1^3-4^10-11^14- , 2^5-6^7-8^9-12^13-15^16-
1	0.01	1-2^3-4^5-6^7-8^9-10^16- , 11-12^13-14^15-
1	0.01	1-2^3-4^10-11^14- , 5-6^7-8^9-12^13-15^16-
1	0.01	0 , 1-2^3-4^5^6-7-8^9-10^11-12^13-14^15-16^
1	0.01	1^3-4^18- , 2^5-6^7-8^9-10^11-12^13-14^15-16^17-
1	0.01	1-2^3-4^5-6^7-8^9-10^11-12^14-15^16-17^18- , 13-
1	0.01	1-2^3-4^5-6^7-8^18- , 9-10^11-12^13-14^15-16^17-
1	0.01	1^5-6^9-10^13-14^15-16^17-18^ , 2^3-4^7-8^11-12^
1	0.01	1-2^3-4^18- , 5-6^7-8^9-10^11-12^13-14^15-16^17-
1	0.01	1^2-3^18- , 4^5-6^7-8^9-10^11-12^13-14^15-16^17-
1	0.01	1^4-5^18- , 2^3-6^7-8^9-10^11-12^13-14^15-16^17-
1	0.01	1-2^3-4^9-10^11-12^17-18^ , 5-6^7-8^13-14^15-16^
1	0.01	1^3-4^5-6^7-8^9-10^11-12^13-14^15-16^17-18^ , 2^
1	0.01	1^5-6^7-8^13-14^15-16^17-18^ , 2^3-4^9-10^11-12^
1	0.01	1-2^3-4^5-6^ , 7-8^9-10^11-12^13-14^15-16^17-18^
1	0.01	1^5-6^18- , 2^3-4^7-8^9-10^11-12^13-14^15-16^17-
1	0.01	1^3-4^5-6^7-8^ , 2^9-10^11-12^13-14^15-16^17-18^
1	0.01	1^5-6^17-18^ , 2^3-4^7-8^9-10^11-12^13-14^15-16^
1	0.01	1^7-8^9-10^11-12^13-14^15-16^17-18^ , 2^3-4^5-6^
1	0.01	1-2^15-16^17-18^ , 3-4^5-6^7-8^9-10^11-12^13-14^
1	0.01	1-3^7-8^18- , 2-4^5-6^9-10^11-12^13-14^15-16^17-
1	0.01	1-2^3-4^5-6^11-12^13-14^ , 7-8^9-10^15-16^17-18^
1	0.01	1-2^3-4^5-6^13-14^15-16^17-18^ , 7-8^9-10^11-12^
1	0.01	1-2^3-4^5-6^7-8^9-10^11-12^17-18^19-20^ , 13-14^15-16^
1	0.01	1-2^3-4^9-10^15-16^20- , 5-6^7-8^11-12^13-14^17-18^19-
1	0.01	1-2^3-4^5-6^7-8^9-10^11-12^13-14^15-16^17-18^19- , 20-
1	0.01	1^ , 2^3-4^5-6^7-8^9-10^11-12^13-14^15-16^17-18^19-20^
1	0.01	1^5-6^7-8^9-10^11-12^13-14^15-16^17-18^19- , 2^3-4^20-
1	0.01	1-2^3-4^9-10^11-12^17-18^ , 5-6^7-8^13-14^15-16^19-20^
1	0.01	1^9-10^ , 2^3-4^5-6^7-8^11-12^13-14^15-16^17-18^19-20^
1	0.01	1-2^3-4^5-6^7-8^9-10^ , 11-12^13-14^15-16^17-18^19-20^
1	0.01	1-2^3-4^13-14^18- , 5-6^7-8^9-10^11-12^15-16^17-19^20-
1	0.01	1^5-6^7-8^11-12^15-16^17-18^19-20^ , 2^3-4^9-10^13-14^
1	0.01	1-2^3-4^22- , 5-6^7-8^9-10^11-12^13-14^15-16^17-18^19-20^21-
1	0.01	1-2^3-4^7-8^9-10^11-12^13-14^15-16^17-18^19-20^21- , 5-6^22-
1	0.01	1^5-6^14-15^22- , 2^3-4^7-8^9-10^11-12^13-16^17-18^19-20^21-
1	0.01	1^13-14^22- , 2^3-4^5-6^7-8^9-10^11-12^15-16^17-18^19-20^21-
1	0.01	1^3-4^22- , 2^5-6^7-8^9-10^11-12^13-14^15-16^17-18^19-20^21-
1	0.01	1^22- , 2^3-4^5-6^7-8^9-10^11-12^13-14^15-16^17-18^19-20^21-
1	0.01	1^ , 2^3-4^5-6^7-8^9-10^11-12^13-14^15-16^17-18^19-20^21-22^
1	0.01	1-2^3-4^5-6^17-18^19-20^21-22^ , 7-8^9-10^11-12^13-14^15-16^23-24^
1	0.01	1^15-16^24- , 2^3-4^5-6^7-8^9-10^11-12^13-14^17-18^19-20^21-22^23-
1	0.01	1-2^3-4^5-6^7-8^9-10^11-12^ , 13-14^15-16^17-18^19-20^21-22^23-24^
1	0.01	1^5-6^26- , 2^3-4^7-8^9-10^11-12^13-14^15-16^17-18^19-20^21-22^23-24^25-
1	0.01	1-2^3-4^5-6^7-8^9-10^11-12^13-14^15-16^17-18^19-20^21-22^23-24^25- , 26-
1	0.01	1-2^3-4^5-6^7-8^9-10^11-12^13-14^15-16^17-18^19-20^21-22^23-24^ , 25-26^
1	0.01	1^3-4^26- , 2^5-6^7-8^9-10^11-12^13-14^15-16^17-18^19-20^21-22^23-24^25-
1	0.01	1^13-14^26- , 2^3-4^5-6^7-8^9-10^11-12^15-16^17-18^19-20^21-22^23-24^25-
1	0.01	1-2^3-4^5-6^7-8^9-10^11-12^13-14^15-16^17-18^19-20^21-22^27- , 23-24^25-26^28-
1	0.01	1-2^3-4^5-6^7-8^9-10^11-12^13-14^ , 15-16^17-18^19-20^21-22^23-24^25-26^27-28^
1	0.01	1^5-6^28- , 2^3-4^7-8^9-10^11-12^13-14^15-16^17-18^19-20^21-22^23-24^25-26^27-

1	0.01	1-2^3-4^5-6^7-8^9-10^11-12^13-14^15-16^17-18^19-20^21-22^23-24^25-26^27-, 28-
1	0.01	1^9-10^30- , 2^3-4^5-6^7-8^11-12^13-14^15-16^17-18^19-20^21-22^23-24^25-26^27-28^29-
1	0.01	1^5-6^30- , 2^3-4^7-8^9-10^11-12^13-14^15-16^17-18^19-20^21-22^23-24^25-26^27-28^29-
1	0.01	1-2^30- , 3-4^5-6^7-8^9-10^11-12^13-14^15-16^17-18^19-20^21-22^23-24^25-26^27-28^29-
1	0.01	0 , 1-2^3-4^5-6^7-8^9-10^11-12^13-14^15-16^17-18^19-20^21-22^23-24^25-26^27-28^29-30^
1	0.01	1-2^3-4^5-6^7-8^9-10^11-12^13-14^15-16^ , 17-18^19-20^21-22^23-24^25-26^27-28^29-30^31-32^
1	0.01	1^21-22^32- , 2^3-4^5-6^7-8^9-10^11-12^13-14^15-16^17-18^19-20^23-24^25-26^27-28^29-30^31-
1	0.01	1-2^3-4^5-6^7-8^9-10^11-12^13-14^15-16^17-18^19-20^21-22^23-24^25-26^27-28^29-30^31- , 32-
1	0.01	1^10-11^32- , 2^3-4^5-6^7-8^9-12^13-14^15-16^17-18^19-20^21-22^23-24^25-26^27-28^29-30^31-
1	0.01	1^32- , 2^3-4^5-6^7-8^9-10^11-12^13-14^15-16^17-18^19-20^21-22^23-24^25-26^27-28^29-30^31-
1	0.01	1^25-26^32- , 2^3-4^5-6^7-8^9-10^11-12^13-14^15-16^17-18^19-20^21-22^23-24^27-28^29-30^31-
1	0.01	1^21-22^ , 2^3^4-5-6^7-8^9-10^11-12^13-14^15-16^17-18^19-20^23-24^25-26^27-28^29-30^31-32^33-34^
1	0.01	1^10-11^34- , 2^3-4^5-6^7-8^9-12^13-14^15-16^17-18^19-20^21-22^23-24^25-26^27-28^29-30^31-32^33-
1	0.01	0 , 1-2^3-4^5-6^7-8^9-10^11-12^13-14^15-16^17-18^19-20^21-22^23-24^25-26^27-28^29-30^31-32^33-34^
1	0.01	1^34^35-36- , 2^3-4^5-6^7-8^9-10^11-12^13-14^15-16^17-18^19-20^21-22^23-24^25-26^27-28^29-30^31-32^33-
1	0.01	1^33-34^35-36^ , 2^3-4^5-6^7-8^9-10^11-12^13-14^15-16^17-18^19-20^21-22^23-24^25-26^27-28^29-30^31-32^
1	0.01	1-2^3-4^5-6^7-8^9-10^11-12^13-14^15-16^17-18^19-20^21-22^23-24^25-26^27-28^29-30^31-32^35-36^ , 33-34^
1	0.01	1^33-34^36- , 2^3-4^5-6^7-8^9-10^11-12^13-14^15-16^17-18^19-20^21-22^23-24^25-26^27-28^29-30^31-32^35-
1	0.01	1^ , 2^3-4^5-6^7-8^9-10^11-12^13-14^15-16^17-18^19-20^21-22^23-24^25-26^27-28^29-30^31-32^33-34^35-36^37-38^
1	0.01	0 , 1-2^3-4^5-6^7-8^9-10^11-12^13-14^15-16^17-18^19-20^21-22^23-24^25-26^27-28^29-30^31-32^33-34^35-36^37-38^
1	0.01	1^ , 2^3-4^5-6^7-8^9-10^11-12^13-14^15-16^17-18^19-20^21-22^23-24^25-26^27-28^29-30^31-32^33-34^35-36^37-38^39-40^
1	0.01	1^18-19^20-21^22-23^24-25^26-27^28-29^34-35^36-37^38-39^ , 2^3-4^5-6^7-8^9-10^11-12^13-14^15-16^17-30^31-32^33-40^
1	0.01	1-2^3-4^5-6^7-8^9-10^11-12^13-14^15-16^17-18^19-20^21-22^23-24^25-26^27-28^29-30^31-32^33-34^35-36^ , 37-38^39-40^41-42^43-44^
1	0.01	1^11-12^44- , 2^3-4^5-6^7-8^9-10^13-14^15-16^17-18^19-20^21-22^23-24^25-26^27-28^29-30^31-32^33-34^35-36^37-38^39-40^41-42^43-
1	0.01	1-2^3-4^5-6^7-8^9-10^11-12^13-14^15-16^17-18^19-20^21-22^23-24^25-26^27-28^29-30^31-32^33-34^37-38^39-40^41-42^43-44^45-46^ , 35-36^
1	0.01	0 , 1-2^3-4^5-6^7-8^9-10^11-12^13-14^15-16^17-18^19-20^21-22^23-24^25-26^27-28^29-30^31-32^33-34^35-36^37-38^39-40^41-42^43-44^45-46^
1	0.01	1^7-8^48- , 2^3-4^5-6^9-10^11-12^13-14^15-16^17-18^19-20^21-22^23-24^25-26^27-28^29-30^31-32^33-34^35-36^37-38^39-40^41-42^43-44^45-46^47-

1	0.01	1-2^3-4^5-6^7-8^9-10^11-12^13-14^15-16^17-18^19-20^21-22^23-24^25-26^27-28^29-30^31-32^33-34^35-36^39-40^41-42^43-44^45-46^47-48^ , 37-38^
1	0.01	0 , 1-2^3-4^5-6^7-8^9-10^11-12^13-14^15-16^17-18^19-20^21-22^23-24^25-26^27-28^29-30^31-32^33-34^35-36^37-38^39-40^41-42^43-44^45-46^47-48^
1	0.01	1-2^3-4^5-6^7-8^18-19^50- , 9-10^11-12^13-14^15-16^17-20^21-22^23-24^25-26^27-28^29-30^31-32^33-34^35-36^37-38^39-40^41-42^43-44^45-46^47-48^49-
1	0.01	0 , 1-2^3-4^5-6^7-8^9-10^11-12^13-14^15-16^17-18^19-20^21-22^23-24^25-26^27-28^29-30^31-32^33-34^35-36^37-38^39-40^41-42^43-44^45-46^47-48^49-50^
1	0.01	0 , 1-2^3-4^5-6^7-8^9-10^11-12^13-14^15-16^17-18^19-20^21-22^23-24^25-26^27-28^29-30^31-32^33-34^35-36^37-38^39-40^41-42^43-44^45-46^47-48^49-50^51-52^
1	0.01	1-2^3-4^5-6^7-8^9-10^11-12^13-14^15-16^17-18^19-20^21-22^23-24^25-26^27-28^29-30^31-32^33-34^37-38^39-40^41-42^43-44^45-46^47-48^49-50^51-52^53-54^55-56^57-58^59-60^61-62^63- , 35-36^64-
1	0.01	1^72- , 2^3-4^5-6^7-8^9-10^11-12^13-14^15-16^17-18^19-20^21-22^23-24^25-26^27-28^29-30^31-32^33-34^35-36^37-38^39-40^41-42^43-44^45-46^47-48^49-50^51-52^53-54^55-56^57-58^59-60^61-62^63-64^65-66^67-68^69-70^71-
