# Supplementary material for: A General Definition and Nomenclature for Alternative Splicing Events
Source: PLoS Comput Biol. 2008 Aug 8;4(8):e1000147. doi: 10.1371/journal.pcbi.1000147 (PMC2467475; doi:10.1371/journal.pcbi.1000147)
Supplement: Table S2 — Medium exon/intron-length in 12 metazoan species. The EnsEmbl annotations for the genomes of the 12 metazoan species have been used to determine the medium exon and intron length (in nt). Introns with non-canonical splice site dinucleotides (i.e., not GT/AG) and exons that are flanked by such have been disregarded for the analysis. Based on these the median exon and intron length has been estimated, that confirms current estimates: whereas there is not much fluctuation in the median exon length, introns are substantially longer in mammals than in other vertebrates, and even shorter in invertebrates. (0.10 MB PDF) [file pcbi.1000147.s002.pdf]

group	species	medium length exons	medium length introns
mammals	human	134	1,525
	chimp	128	1,504
	mouse	134	1,250
	rat	125	1,085
	dog	121	1,006
	cow	120	969
lower vertebrates	chicken	126	816
	frog	121	855
	zebrafish	118	704
In-vertebrates	honeybee	125	159
	fruitfly	274	82
	worm	157	67
