# Supplementary material for: A General Definition and Nomenclature for Alternative Splicing Events
Source: PLoS Comput Biol. 2008 Aug 8;4(8):e1000147. doi: 10.1371/journal.pcbi.1000147 (PMC2467475; doi:10.1371/journal.pcbi.1000147)
Supplement: Table S3 — Attributes of the transcriptome in 12 metazoan species. For each of the 12 species under analysis, this table shows the number of loci (according to the transcript clustering described herein) and the number of transcripts in the corresponding EnsEmbl annotation. Next, the number of variations in the exon-intron structure detected by our method is reported and the subgroup of them that conforms with the requirements for an AS event (Definition 4), exhibits canonical GT/AG splice site dinucleotides and does not involve alternative transcription start/poly-adenylation sites. Finally, the average number of exons per locus that are flanked by canonical GT/AG splice sites is given with the respective standard-deviation across the genome. (0.24 MB PDF) [file pcbi.1000147.s003.pdf]

	loci	transcripts	variations	AS event (GT/AG)	exons (GT/AG) per gene
human	22,303	43,102	59,676	12,206	8.5 ± 11.2
chimp	23,326	36,203	37,856	8,444	8.8 ± 10.7
mouse	27,133	35,622	24,921	4,062	7.9 ± 10.2
rat	24,764	37,657	39,071	6,150	8.2 ± 11.4
dog	21,856	28,672	16,521	2,804	8.2 ± 9.8
cow	18,574	26,930	22,567	1,438	9.1 ± 11.6
chicken	15,568	21,140	16,308	1,402	10.0 ± 10.8
frog	20,527	29,430	59,476	3,258	8.4 ± 9.6
zebrafish	27,015	38,290	49,755	3,611	7.7 ± 9.2
honeybee	12,949	27,841	83,843	1,676	5.3 ± 6.8
fruitfly	13,751	20,040	12,382	2,970	4.6 ± 4.6
worm	20,761	27,486	16,925	1,575	6.5 ± 5.0
