# Supplementary material for: A General Definition and Nomenclature for Alternative Splicing Events
Source: PLoS Comput Biol. 2008 Aug 8;4(8):e1000147. doi: 10.1371/journal.pcbi.1000147 (PMC2467475; doi:10.1371/journal.pcbi.1000147)
Supplement: Figure S1 — UCSC genome browser screenshots for 5 AS events. Screenshots of UCSC genome browser depicting the AS events discussed in Figure 1 in the genes VEGFA (A), CLEC10A (B), TCL6 (C), AURKC (D), and AIF1 (E). Blue boxes are exons, with the coding regions visualized as thicker areas. Chromosomal coordinates and RefSeq identifiers are given to the top respectively to the left. (0.31 MB PDF) [file pcbi.1000147.s004.pdf]

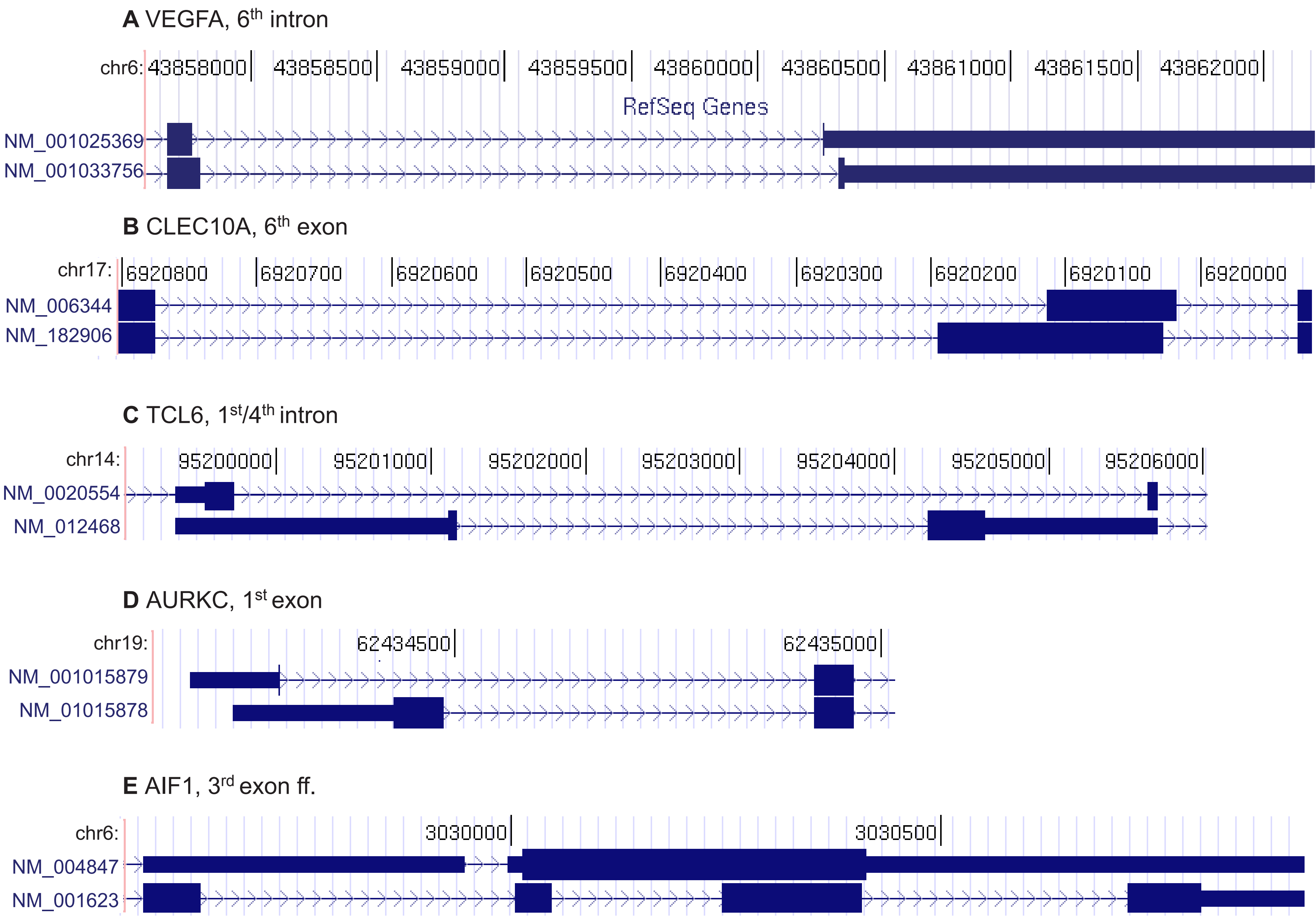
A VEGFA, 6th intron
chr6: 43858000 43858500 43859000 43859500 43860000 43860500 43861000 43861500 43862000
RefSeq Genes
NM_001025369
NM_001033756
B CLEC10A, 6th exon
chr17: 6920800 6920700 6920600 6920500 6920400 6920300 6920200 6920100 6920000
NM_006344
NM_182906
C TCL6, 1st/4th intron
chr14: 95200000 95201000 95202000 95203000 95204000 95205000 95206000
NM_0020554
NM_012468
D AURKC, 1st exon
chr19: 62434500 62435000
NM_001015879
NM_01015878
E AIF1, 3rd exon ff.
chr6: 3030000 3030500
NM_004847
NM_001623
