# Supplementary material for: A General Definition and Nomenclature for Alternative Splicing Events
Source: PLoS Comput Biol. 2008 Aug 8;4(8):e1000147. doi: 10.1371/journal.pcbi.1000147 (PMC2467475; doi:10.1371/journal.pcbi.1000147)

FOXP2

A AStalavista

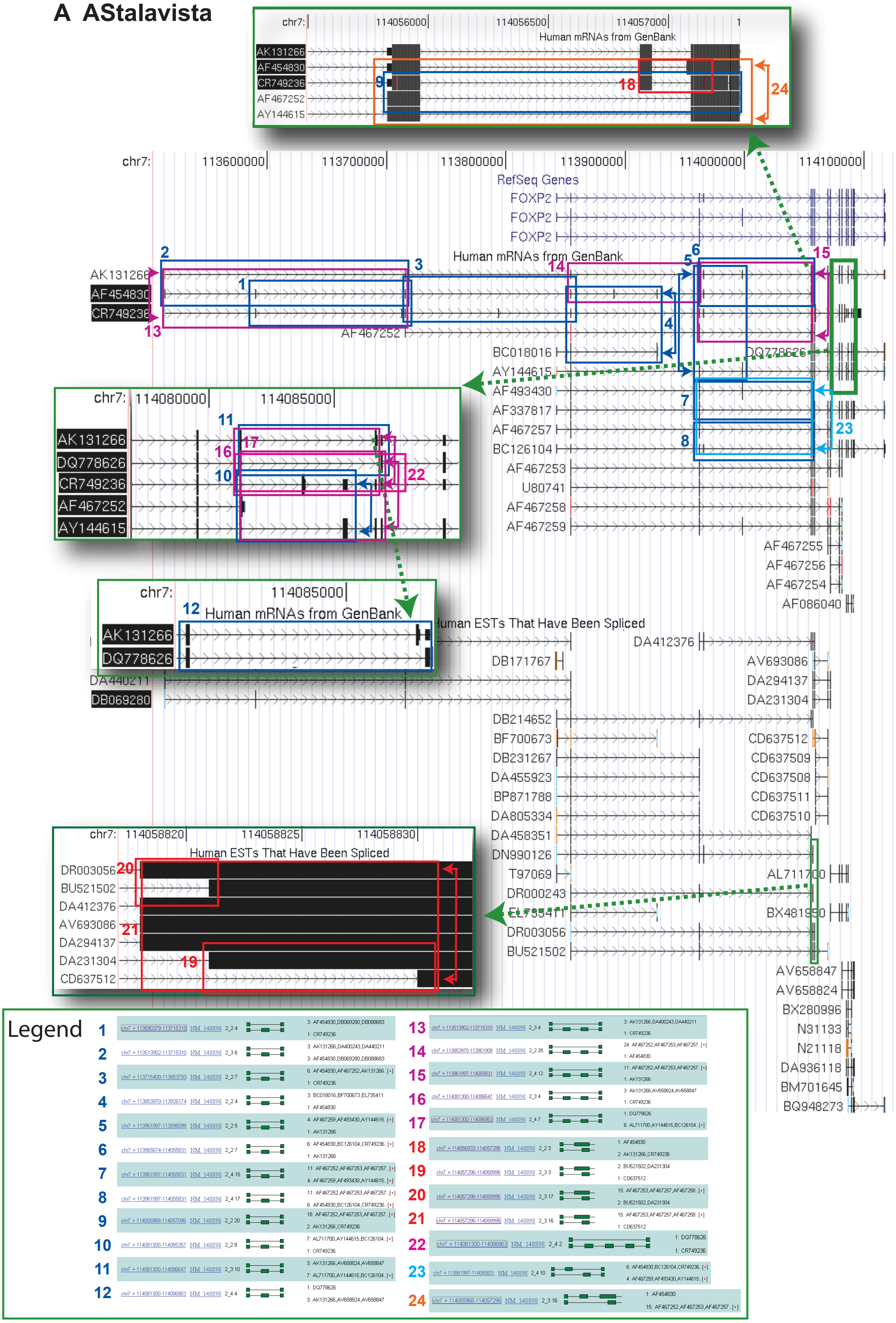

B Hollywood

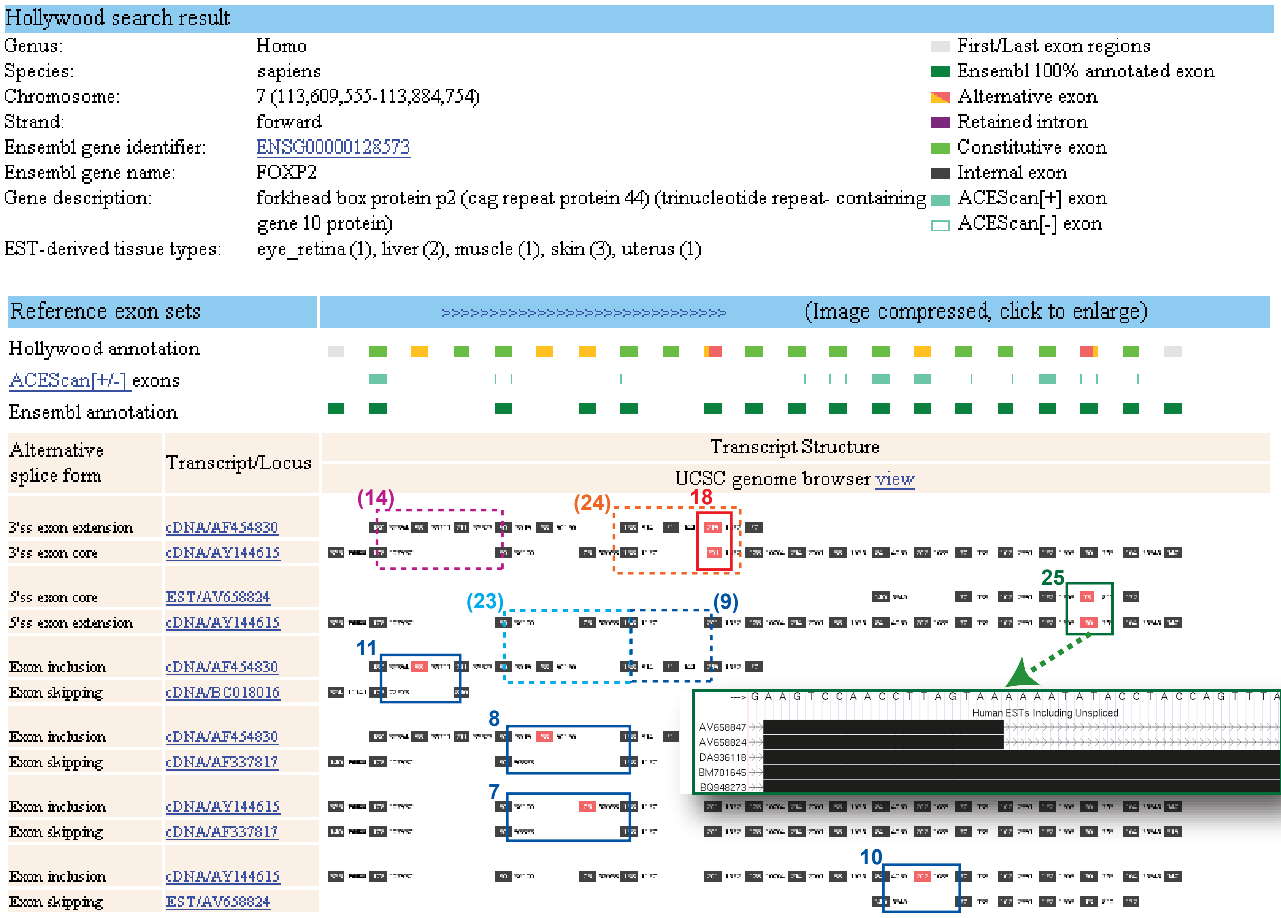

C EuSplice

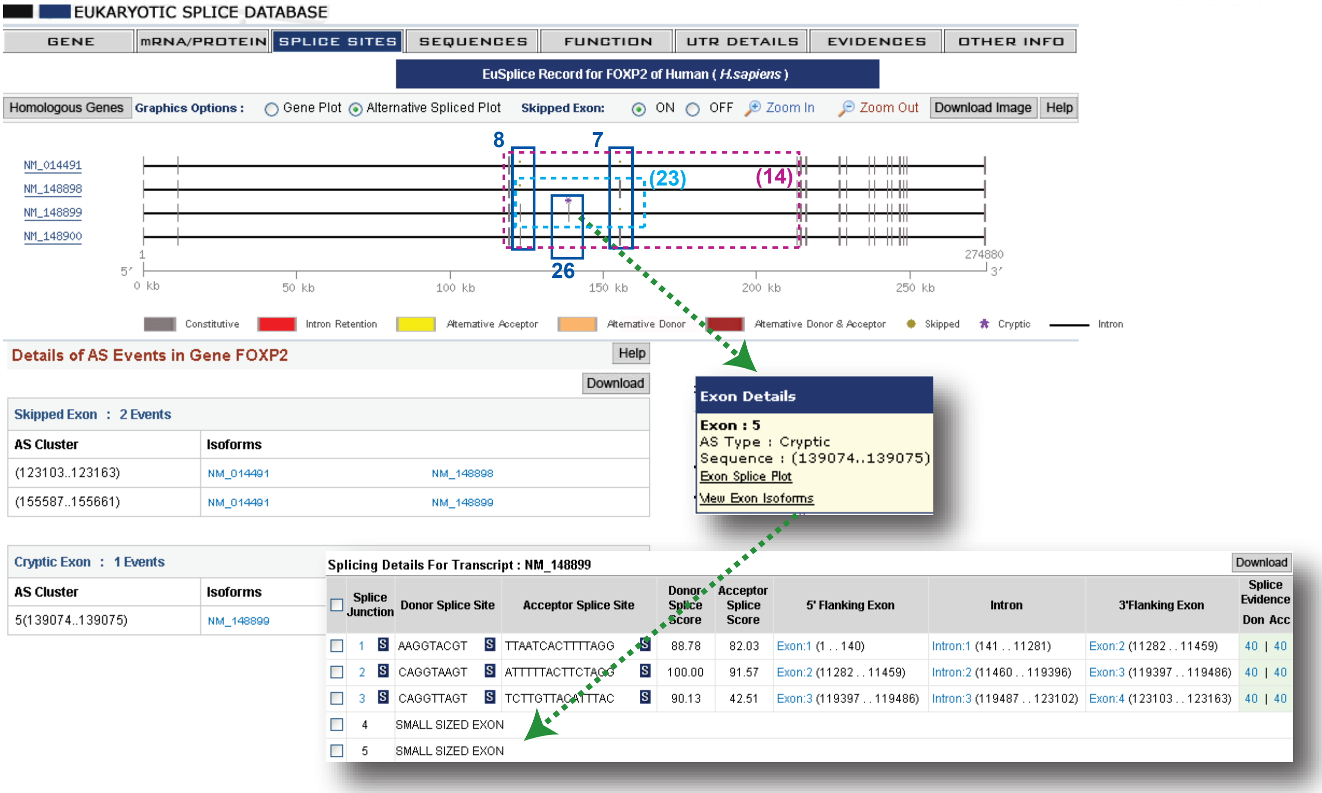

Supplement: Figure S2 — AS events in the FOXP2 gene. Exploded assembly drawing of the AS events found by AStalavista (A), Hollywood (B), and EuSplice (C) in the FOXP2 gene. The region of events is outlined by a rectangle and double arrows indicate the pairwisely compared variants. The events are numbered consecutively and colors mark different structures: 0,1–2ˆ is blue (events 1–12 and 26), 0,1–2ˆ3–4ˆ is purple (events 13–17), 1-,2- is red (events 18–20), 0,1–2ˆ3–4ˆ5–6ˆ is pink (event 22), 1–2ˆ,3–4ˆ is electric blue (event 23), 1–2ˆ3-,4- is orange (event 24). Hollywood shows splice donor variation (event 25) that is not found by AStalavista since it exhibits the unusual splice donor sequence AAAAT. EuSplice predicts additionally event 26, a cryptic exon that has been inferred from a 2 nt alignment of the mRNA sequence to the genome. In contrast, AStalavista finds 8 more bona fide events with mRNA support than EuSplice and 19 more events in ESTs than Hollywood. (0.26 MB PDF) [file pcbi.1000147.s005.pdf]
