# Supplementary figures and images for: A General Definition and Nomenclature for Alternative Splicing Events
Source: PLoS Comput Biol. 2008 Aug 8;4(8):e1000147. doi: 10.1371/journal.pcbi.1000147 (PMC2467475; doi:10.1371/journal.pcbi.1000147)

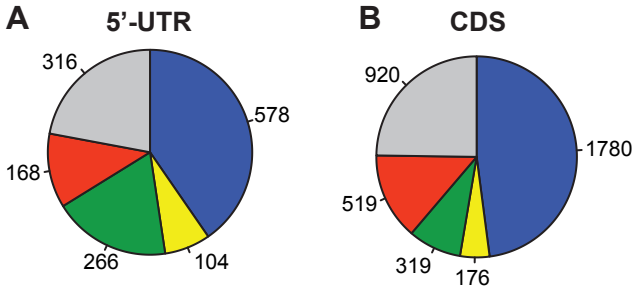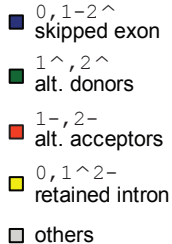

Supplement: Figure S3 — Formed by AS events overlapping the 5′ UTR/CDS. Pie diagrams depicting the landscape of AS events in the RefSeq annotation that are overlapping the respective 5′UTR (A) or the CDS (B) of coding transripts. Qualitatively the same trends can be observed as in Figure 4, events overlapping the CDS show relatively more alternative exons, less alternative introns and much less splice donor variance compared to the acceptor variance. (0.27 MB PDF) [file pcbi.1000147.s006.pdf]

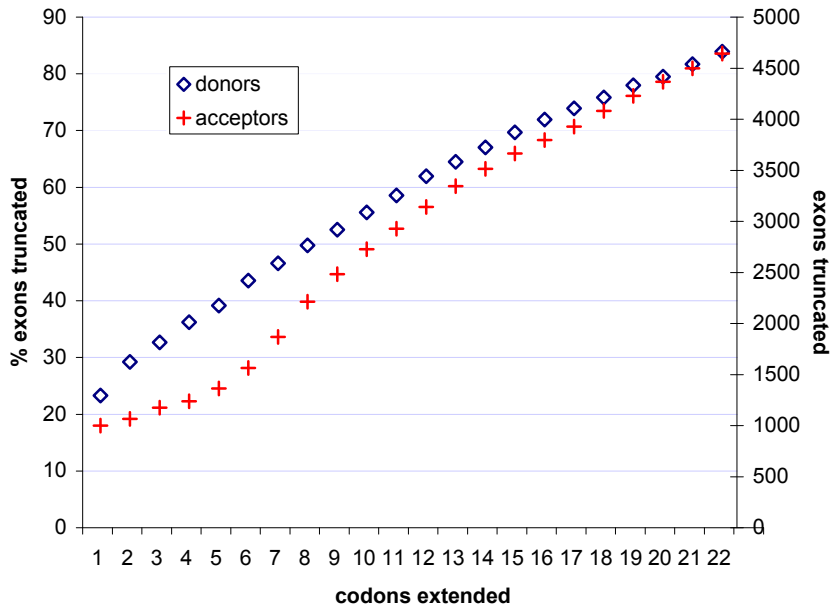

Supplement: Figure S4 — Cumulative exon truncation at the splice donor/acceptor. The plot shows the cumulative curve for the data presented in Figure 5: hypothetical truncations of the annotated CDSs when extending artificially a certain number of codons (horizontal axis) into the intron from the splice donor (blue diamonds) and acceptor (red crosses) of coding exons. Up to 22 codons of extension, the profile of the splice site sequence causes more exons to be truncated when adopting intronic sequence at the splice donor site. (0.15 MB PDF) [file pcbi.1000147.s007.pdf]

**A CDS**

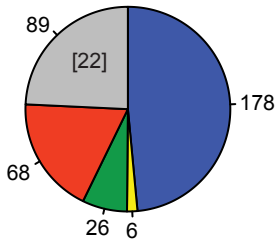

**B non-coding transcripts**

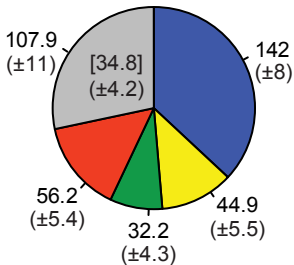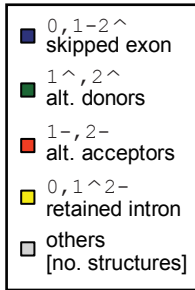

Supplement: Figure S5 — AS landscape in random subsets of noncoding transcripts. In order to compare the landscape of AS events located in CDSs of coding transcripts (A) with the landscape formed by events in non-coding transcripts (B) in equally sized sets (see Figure 6), 100 datasets of 1,332 noncoding transcripts have been randomly sampled (from the total of 2,247 currently annotated in Gencode) and analyzed. The number of events is presented (arithmetic mean with standard deviation in parenthesis for the 100 random datasets of non-coding transcripts) in structurally different groups (colored according to Figure 3). (0.30 MB PDF) [file pcbi.1000147.s008.pdf]
